# The protein aggregation inhibitor YAT2150 has potent antimalarial activity in *Plasmodium falciparum *in vitro cultures

**DOI:** 10.1186/s12915-022-01374-4

**Published:** 2022-10-22

**Authors:** Inés Bouzón-Arnáiz, Yunuen Avalos-Padilla, Arnau Biosca, Omar Caño-Prades, Lucía Román-Álamo, Javier Valle, David Andreu, Diana Moita, Miguel Prudêncio, Elsa M. Arce, Diego Muñoz-Torrero, Xavier Fernàndez-Busquets

**Affiliations:** 1 Barcelona Institute for Global Health (ISGlobal, Hospital Clínic-University of Barcelona), Rosselló 149-153, 08036 Barcelona, Spain; 2 Nanomalaria Group, Institute for Bioengineering of Catalonia (IBEC), The Barcelona Institute of Science and Technology, Baldiri Reixac 10-12, 08028 Barcelona, Spain; 3 Nanoscience and Nanotechnology Institute (IN2UB), University of Barcelona, Martí i Franquès 1, 08028 Barcelona, Spain; 4 Department of Medicine and Life Sciences, Barcelona Biomedical Research Park, Pompeu Fabra University, Dr. Aiguader 88, 08003 Barcelona, Spain; 5 Instituto de Medicina Molecular, Fac. Medicina Univ. Lisboa, Av. Prof. Egas Moniz, 1649-028 Lisbon, Portugal; 6 Laboratory of Medicinal Chemistry (CSIC Associated Unit), Faculty of Pharmacy and Food Sciences, and Institute of Biomedicine (IBUB), University of Barcelona, Av. Joan XXIII, 27-31, 08028 Barcelona, Spain

**Keywords:** *Plasmodium falciparum*, Protein aggregation, YAT2150, Amyloid pan-inhibitors, Malaria, Antimalarial drugs

## Abstract

**Background:**

By 2016, signs of emergence of *Plasmodium falciparum* resistance to artemisinin and partner drugs were detected in the Greater Mekong Subregion. Recently, the independent evolution of artemisinin resistance has also been reported in Africa and South America. This alarming scenario calls for the urgent development of new antimalarials with novel modes of action. We investigated the interference with protein aggregation, which is potentially toxic for the cell and occurs abundantly in all *Plasmodium* stages, as a hitherto unexplored drug target in the pathogen.

**Results:**

Attempts to exacerbate the *P. falciparum* proteome’s propensity to aggregation by delivering endogenous aggregative peptides to in vitro cultures of this parasite did not significantly affect their growth. In contrast, protein aggregation inhibitors clearly reduced the pathogen’s viability. One such compound, the bis(styrylpyridinium) salt YAT2150, exhibited potent antiplasmodial activity with an in vitro IC_50_ of 90 nM for chloroquine- and artemisinin-resistant lines, arresting asexual blood parasites at the trophozoite stage, as well as interfering with the development of both sexual and hepatic forms of *Plasmodium*. At its IC_50_, this compound is a powerful inhibitor of the aggregation of the model amyloid β peptide fragment 1-40, and it reduces the amount of aggregated proteins in *P. falciparum* cultures, suggesting that the underlying antimalarial mechanism consists in a generalized impairment of proteostasis in the pathogen. YAT2150 has an easy, rapid, and inexpensive synthesis, and because it fluoresces when it accumulates in its main localization in the *Plasmodium* cytosol, it is a theranostic agent.

**Conclusions:**

Inhibiting protein aggregation in *Plasmodium* significantly reduces the parasite’s viability in vitro. Since YAT2150 belongs to a novel structural class of antiplasmodials with a mode of action that potentially targets multiple gene products, rapid evolution of resistance to this drug is unlikely to occur, making it a promising compound for the post-artemisinin era.

**Supplementary Information:**

The online version contains supplementary material available at 10.1186/s12915-022-01374-4.

## Background

The available arsenal of antimalarial drugs is insufficient to progress towards eradication of the disease, a scenario that is worsened by the rampant evolution of resistance by *Plasmodium*. This situation calls for immediate efforts to discover new antimalarials of easy and cost-affordable production, having several molecular targets in the pathogen and acting through new antiparasitic mechanisms not shared by currently used drugs.

The deadliest species of the malaria parasite, *Plasmodium falciparum*, is exceptionally rich in proteins containing long glutamine/asparagine (Q/N) repeats [1], which are low complexity regions with a propensity to form insoluble intracellular aggregates [2]. It has been recently reported that protein aggregation occurs abundantly in all *Plasmodium* stages in both vertebrate and mosquito hosts [3]. Because the presence of protein deposits (either amorphous aggregates, large amyloid fibrils, or small soluble oligomers with little or no fibrillar content) within a cell is generally associated with cellular stress and toxicity [4], this distinctive phenotype of malaria parasites could potentially be harnessed to develop new therapeutic strategies based on the perturbation of the pathogen’s proteostasis. Protein aggregates expose hydrophobic residues and unpaired polypeptide backbone structures that interact promiscuously with other molecules and critical factors of the proteostasis network [5]. Moreover, large intracellular deposits can displace membrane structures and may cause their breakdown [6]. As a result, protein aggregation is usually considered toxic for the organism undergoing it. Indeed, proteinaceous assemblies such as amyloids and prions were initially discovered in neurodegenerative diseases and were quickly attributed to an anomalous state of otherwise properly folded proteins. Many pathologies are related to a defect in protein folding and the ensuing aggregation of partially folded intermediates, including Alzheimer’s [7], Parkinson’s [8], and Huntington’s disease [9], amyotrophic lateral sclerosis [10], transmissible spongiform encephalopathies like Creutzfeldt-Jakob disease [11] and scrapies [12], and spinocerebellar ataxia [13]. The cytotoxicity of protein aggregation has also been identified at the root of the evolutionary tree, and bacterial susceptibility to protein misfolding has been proposed as the target of future antibiotics [14].

However, a deeper study of the aggregation process of many amyloids and prion-like proteins has shown that, in some cases, protein aggregation is not associated with a toxic process. Examples of functional amyloids can be found in prokaryotes, where they can be involved in virulence, extracellular matrix assembly, and biofilm formation [15]. In higher organisms, functional amyloids are present in the eggshell of some insects and fish [16, 17] and as a component of silk [18]. In humans, amyloids participate in the storage of peptide hormones [19] and melanin polymerization [20], and have also been proposed to play a role in long-term memory potentiation [21]. Functional roles in malaria parasites for Q/N repeats have been suggested as tRNA sponges [22] and in immune evasion and antigenic variation [23].

Intriguingly, some commonly used antimalarial drugs have been reported to affect protein aggregation. For instance, artemisinin resistance is associated with an increased expression of genes involved in the unfolded protein response [24], in agreement with the hypothesis that artemisinin’s antimalarial activity damages proteins and inhibits the proteasome [25]. Inhibition of the *P. falciparum* proteasome has been shown to have potent gametocytocidal activity [26, 27], suggesting that impairing protein disposal is deleterious for the parasite as a result of the potential toxicity of an increase in unfolded, aggregation-prone proteins. On the other hand, a significant antimalarial drug-based body of evidence hints at the striking possibility that protein aggregation might be functional for malaria parasites. Oligomerization of the amyloid β (Aβ) peptide is inhibited by methylene blue and curcumin [28, 29], and the latter compound also prevented amyloid fibril formation, while another antimalarial, quercetin, showed potent anti-Aβ peptide aggregation activity [30]. A number of quinoline antimalarials (e.g., quinine, chloroquine, amodiaquine, quinacrine, mefloquine, and primaquine) have been reported to inhibit scrapie-associated prion protein accumulation both in vitro [31] and inside cells [32–37]. Quinacrine directly dissociated amyloid plaques in the brain of a 5XFAD transgenic mouse model of Alzheimer’s disease [38]. Certain 4-aminoquinoline-based heterodimeric compounds with antiplasmodial activity in the µM range [39] are strong amyloid pan-inhibitors [40]. Rapamycin, recently shown to reduce the in vitro growth of *P. falciparum* with a half maximal inhibitory concentration (IC_50_) around 2 µM [41], was previously described to decrease protein aggregation in vivo by stimulating autophagy [42] and through protein synthesis inhibition [43, 44]. Also, part of the alleged antimalarial properties of green tea have been tracked down to the flavonoid epigallocatechin-3-gallate [45], which disaggregates the amyloid fibrils formed by the intrinsically unstructured merozoite surface protein 2 [46], a component of the coat present on the *P. falciparum* stage that invades a naïve red blood cell (RBC).

Given this proliferation of results pointing at both stimulation and inhibition of protein aggregation being deleterious for malaria parasites, we have explored here both hypotheses.

## Results

### Effect of endogenous aggregative peptides on *P. falciparum* cultures

Previous studies have established that seeding exacerbates protein aggregation reactions [47] and that homologous seeding is much more efficient than heterologous seeding [48]. Considering the potential cellular toxicity of protein aggregation, we set out to test the hypothesis that endogenous *Plasmodium* peptides with high aggregative capacity could behave as nucleating agents and further increase the already high protein aggregation inside the parasite, which might compromise its viability. In our previous work, 0.1% sodium dodecyl sulfate (SDS)-resistant protein aggregates obtained from late-stage *P. falciparum*-parasitized RBC (pRBC) culture homogenates had been analyzed by liquid chromatography with tandem mass spectrometry (LC–MS/MS) [3]. From the pool of 369 proteins identified, 10 peptides with high aggregation propensity were selected (Additional file 1: Table S1). When incorporated to in vitro pRBC cultures, none of the peptides had a significant effect on parasite growth up to concentrations ≥ 125 µM.

To improve the likely poor entry of aggregative peptides into pRBCs, the 6 sequences from Additional file 1: Table S1 that exhibited the highest tendency to form amyloid fibrils in vitro according to thioflavin T (ThT) fluorescence and transmission electron microscopy (TEM) imaging ([3] and Additional file 1: Fig. S1) were elongated at their N-terminus by the cell-penetrating peptides (CPPs) TP2 (PLIYLRLLRGQF), LMWP (VSRRRRRRGGRRRR), and TAT (GRKKRRQRRRPQ). The peptides were also labeled with fluorescein to allow their detection inside target cells. According to preliminary flow cytometry data, the CPPs by themselves did not show a significant entry into non-infected RBCs (Additional file 1: Figs. S2-S4), despite all three peptides having been described to enter cells by non-endocytic mechanisms [49–51], although intake increased between 3- and 6- fold for pRBCs. Aggregative peptides conjugated to TAT resulted in a cell entry into pRBCs generally lower than for TAT alone, whereas conjugation to LMWP and TP2 increased in most cases the intake by pRBCs. Penetration into non-parasitized erythrocytes remained roughly constant for LMWP- and TAT-elongated peptides but decreased for TP2-conjugated peptides relative to free TP2. The best results were obtained using TP2, with TP2-LQSNIG entering 17% of pRBCs. Peptide intake was mainly in late erythrocytic *Plasmodium* stages, in agreement with the observed lack of penetration into naïve RBCs. Late forms are known to have an increased permeability to extracellular components [52], suggesting that it was this characteristic, rather than CPP activity, the main drive to make CPP-aggregative peptides enter pRBCs.

**Fig. 1:**
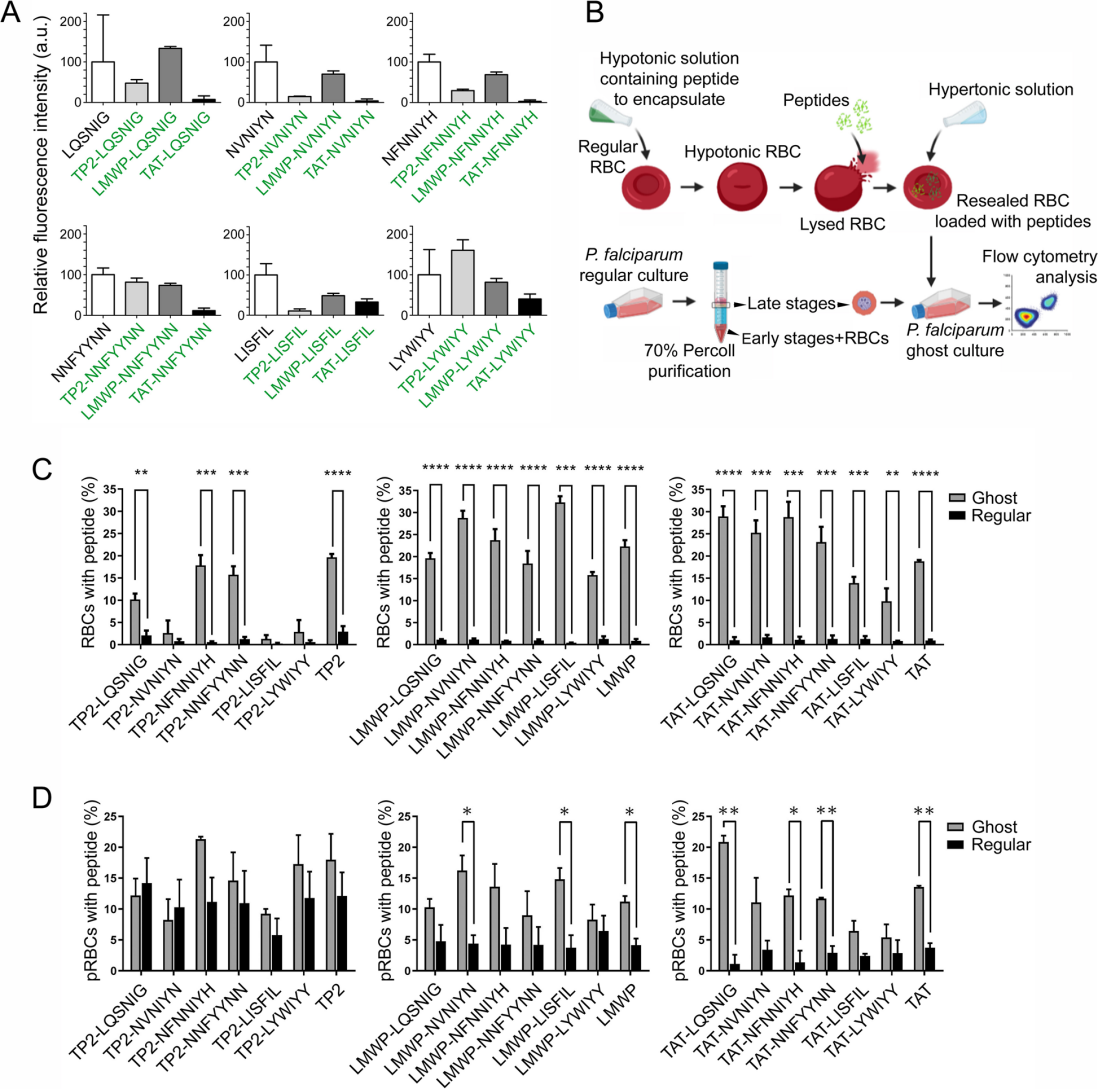
Loading of aggregative peptides into ghost RBCs. **A** ProteoStat® in vitro aggregation assay of aggregative peptides conjugated to fluorescein-labeled CPPs. The control normalized to 100% aggregation corresponds to the unconjugated, fluorescein-free aggregative peptides. a.u.: arbitrary units. Graphs show the mean ± SEM of three independent experiments. **B** Scheme of peptide loading into *P. falciparum* ghosts. **C**, **D** Flow cytometry analysis of the targeting of fluorescein-labeled CPP-conjugated aggregative peptides in ghost and regular RBC suspensions. **C** Fraction of RBCs positive for fluorescein-labeled peptides. **D** Colocalization analysis of *Plasmodium* and fluorescein-labeled peptides 72 h post-infection. The parasitemias achieved in all cases were within the expected values for regular *P. falciparum* cultures. Bars represent the means ± SEM of at least two independent experiments where 30,000 events were recorded in the flow cytometer. *: *p* ≤ 0.05; **: *p* ≤ 0.01; ***: *p* ≤ 0.001; ****: *p* ≤ 0.0001

Conjugation of self-aggregative peptides to fluorescein-labeled CPPs had different effects on their in vitro aggregation capacity (Fig. 1A), which generally decreased but remained relevant in most cases. Conjugation to TAT resulted in the most significant drop in aggregation, with only TAT-LISFIL and TAT-LYWIYY retaining > 30% of their self-aggregative potential. On the other hand, LMWP was the least intrusive CPP in this regard, whereby only LMWP-LISFIL showed a clear drop of ca. 50% aggregation relative to CPP-free LISFIL. Conjugation to TP2 offered the most disperse results, with some peptides like TP2-NVNIYN and TP2-LISFIL losing most of their aggregative capacity and others such as TP2-LYWIYY clearly increasing it. When incorporated to in vitro* P. falciparum* growth inhibition assays, CPP-conjugated aggregative peptides did not have a significant impact on the viability of the parasite unless very high amounts were used (Additional file 1: Table S2). Peptide concentrations up to 200 µM resulted in parasitemias similar to those of untreated controls, the best result being obtained with some LMWP-elongated peptides, which at 20 µM induced a modest growth inhibition around 10%.

To further increase the presence inside pRBCs of endogenous *P. falciparum* aggregative peptides, a previously described protocol for the transfection of ghost RBCs [53] was adapted to preload these with peptides prior to *Plasmodium* infection (Fig. 1B). In our in vitro* P. falciparum* culture conditions, ghosts were successfully invaded by the parasite, which could grow inside them at a rate undistinguishable from assays where intact RBCs were used as host cells (Additional file 1: Fig. S5). Aggregative peptides conjugated to fluorescein-labeled CPPs exhibited in general a better intake by ghost RBC suspensions relative to regular RBCs (Fig. 1C), except for some TP2-conjugated peptides, whose entry into ghosts remained low. These peptide-loaded RBCs could be infected by *P. falciparum*, being the proportion of peptide-containing pRBCs significantly larger in ghost-enriched cultures, especially for TAT- and LMWP-conjugated peptides (Fig. 1D). Overall, using ghosts as host cells resulted in between 5 and 20% of *Plasmodium*-infected cells containing exogenously added peptides as determined by flow cytometry analysis, which was deemed sufficient to assess the effect on parasite growth of peptide-treated samples relative to untreated controls.

When incorporated at 10 µM into ghost RBC preparations to be used for in vitro* P. falciparum* growth inhibition assays, LMWP-conjugated aggregative peptides had the largest impact on the viability of the parasite (Table 1). This result was consistent with the observations that LMWP had a good entrance in ghosts (Fig. 1C) and that it was the least disruptive CPP for the aggregation of *Plasmodium* peptides (Fig. 1A). LMWP-NFNNIYH was the most active peptide, reducing *Plasmodium* growth to ca. 66% that of the untreated control. Among the other CPP-conjugated peptides, only TP2-LYWIYY had a significant effect leading to a ca. 85% parasite growth relative to the control. Remarkably, some CPP-free and fluorescein-free aggregative peptides did also decrease parasite viability in ghost-pRBC cultures, such as GLVFFI and YLFFIS, which resulted, respectively, in 79.4 ± 0.9% and 77.5 ± 12.3% growth relative to the untreated control (Additional file 1: Table S3). These two peptides had a relatively low cytotoxicity in human umbilical vein endothelial cells cultures (Additional file 1: Table S4), suggesting that their effect on *P. falciparum* might represent a genuine antiplasmodial activity.

**Table 1:** Growth inhibition assay in the *P. falciparum* cultures done in ghost RBC preparations treated with 10 µM fluorescein-labeled aggregative peptides conjugated to CPPs from Fig. 1D

Peptide sequence	Parasite growth relative to untreated control (%) ± SEM	*p*-value^1^
TAT	100.0 ± 0.9	> 0.9999
TAT-LQSNIG	100.0 ± 17.5	> 0.9999
TAT-NVNIYN	99.7 ± 5.2	0.9580
TAT-NFNNIYH	100.0 ± 10.8	> 0.9999
TAT-NNFYYNN	100.0 ± 6.1	> 0.9999
TAT-LISFIL	95.5 ± 6.4	0.5572
TAT-LYWIYY	100.0 ± 5.9	> 0.9999
TP2	100.0 ± 9.5	> 0.9999
TP2-LQSNIG	100.0 ± 4.2	> 0.9999
TP2-NVNIYN	100.0 ± 12.0	> 0.9999
TP2-NFNNIYH	100.0 ± 7.3	> 0.9999
TP2-NNFYYNN	96.2 ± 0.3	0.7000
TP2-LISFIL	98.5 ± 0.5	0.0835
TP2-LYWIYY	84.9 ± 3.0	0.0374*
LMWP	90.9 ± 1.1	0.3430
LMWP-LQSNIG	75.2 ± 3.4	0.0188*
LMWP-NVNIYN	81.9 ± 10.1	0.2152
LMWP-NFNNIYH	66.2 ± 6.5	0.0349*
LMWP-NNFYYNN	73.6 ± 12.4	0.1665
LMWP-LISFIL	79.4 ± 9.3	0.1561
LMWP-LYWIYY	86.3 ± 6.4	0.1653

^1^*p*-value obtained applying two-sided Student’s *t* test in GraphPad Prism. *p ≤ 0.05

**Fig. 2:**
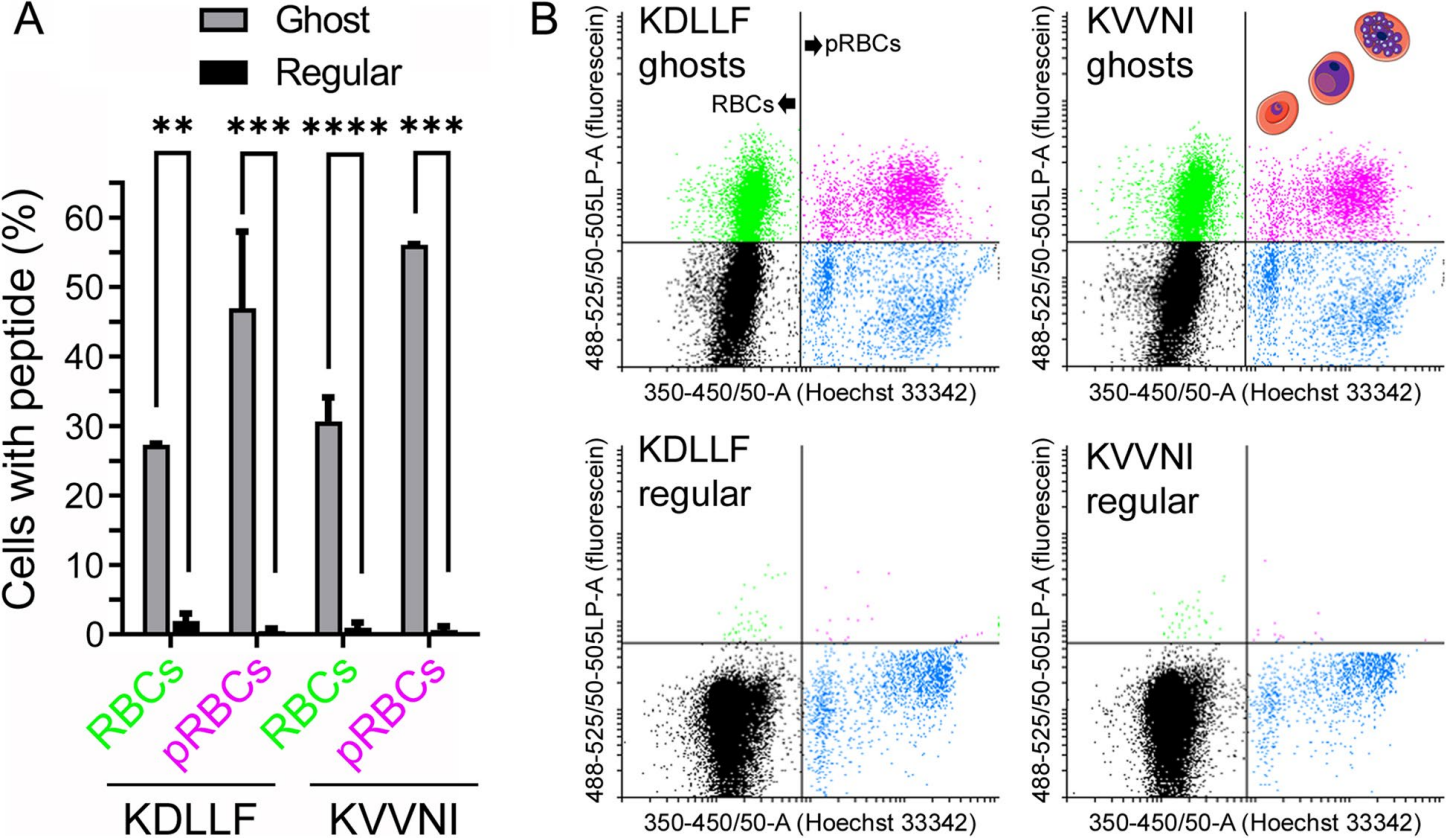
Flow cytometry analysis of the presence of the fluorescein-labeled peptides KDLLF and KVVNI in ghost RBCs. **A** Bar graph of the aggregated data. Bars represent the means ± SEM of at least two independent experiments where 30,000 events were recorded in the flow cytometer. **: *p* ≤ 0.01; ***: *p* ≤ 0.001; ****: *p* ≤ 0.0001. **B** Representative examples of flow cytometry plots to illustrate for both peptides their presence inside ghost vs. regular RBCs and pRBCs. The cartoons in the upper right plot roughly indicate from left to right the cell populations corresponding to ring, trophozoite, and schizont forms

As mentioned above, the 10 peptides chosen for this proof-of-concept study of the potential toxicity of endogenous protein aggregation for *Plasmodium* were selected from late-stage cultures. This form of the pathogen has grown to completely fill the host RBC cytosol and therefore contains the highest amount of parasite protein of all the blood stages, which did facilitate the identification of a larger number of potentially active aggregative peptides. However, this strategy might target proteins that are expressed late in the intraerythrocytic cycle, and therefore the induction of aggregation could occur when the parasite is about to egress its host cell, minimizing the potential antiplasmodial effect of uncontrolled protein aggregation. To identify aggregative peptides in proteins expressed early in the blood cycle, we isolated protein aggregates from early-stage pRBC culture homogenates resisting dissolution in the presence of 0.1% SDS. LC–MS/MS analysis of this sample resulted in the identification of 33 *Plasmodium* proteins (Additional file 1: Table S5), of which 23 were also present in the pool of 369 proteins isolated from late blood stages [3]. These 23 proteins were not highly aggregative, but they were relatively abundant in the parasite (Additional file 1: Fig. S6). Of the 23 proteins analyzed for their abundance and aggregation propensity, the most aggregation-prone was E3 ubiquitin-protein ligase (Uniprot ID: C0H4K6). When this large protein (3893 amino acids) was run through the WALTZ algorithm [54], which identifies amyloid-forming amino acid sequences, 46 peptides with high aggregation propensity were identified (Additional file 1: Table S6). Two of these, KDLLF and KVVNI (WALTZ aggregation scores 96.32 and 96.99, respectively), formed amorphous aggregates in vitro (Additional file 1: Fig. S7) and were present in 10 and 9 *P. falciparum* proteins, respectively (Additional file 1: Table S7). Because aggregation-prone regions can promote the aggregation of the proteins containing them [55], peptides present in early blood stages and in a large number of *Plasmodium* proteins are likely to stimulate protein aggregation for a longer time and over a wider fraction of the proteome. Regular RBCs were essentially impermeable to fluorescein-labeled KDLLF and KVVNI, which however were found in a significant fraction of ghost RBCs in non-parasitized and, especially, in *P. falciparum*-parasitized cultures (Fig. 2). In ghost pRBCs, these exogenously added fluorescein-labeled peptides localized inside the parasitophorous vacuole, and not in the RBC cytosol (Additional file 1: Fig. S8). Within the vacuole, the peptides distributed in both the parasite’s cytosol and in the digestive vacuole, according to confocal fluorescence microscopy colocalization data with markers of these two cellular compartments. However, in in vitro growth inhibition assays done in peptide-containing ghost pRBC preparations, parasite viability was not significantly impaired by either peptide up to a concentration of 10 µM (Additional file 1: Table S8).

### Effect of amyloid pan-inhibitors on *P. falciparum* cultures

**Fig. 3:**
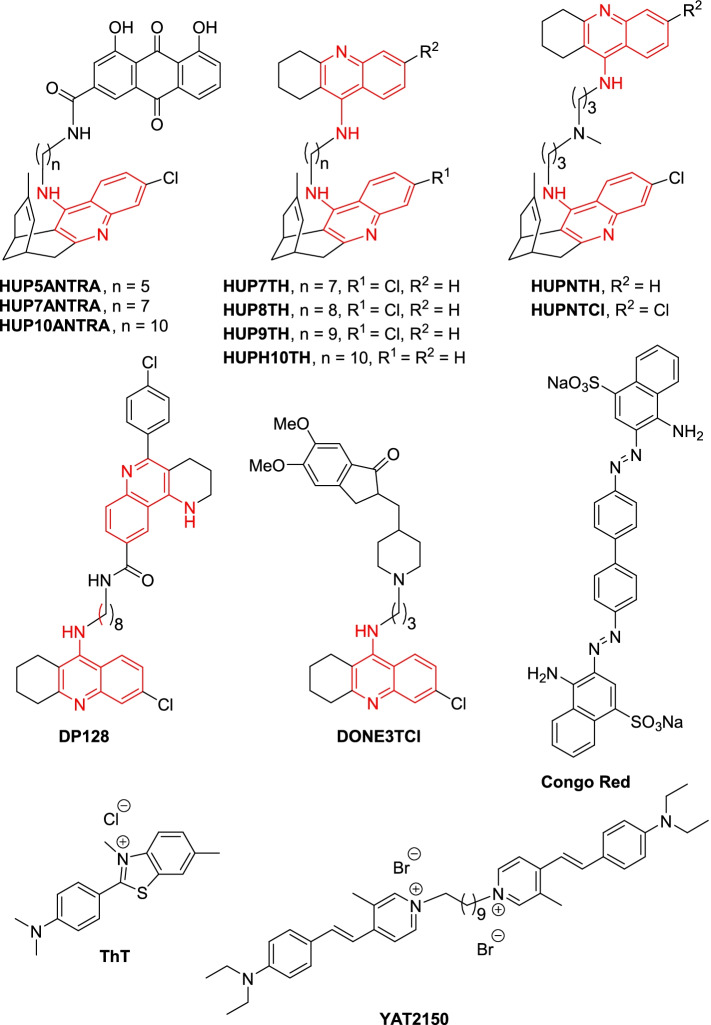
Chemical structures of amyloid pan-inhibitors and β-sheet intercalators that were tested for in vitro antimalarial activity. The 4-aminoquinoline scaffold that is present in amyloid pan-inhibitors is colored in red

The failure to clearly reduce *Plasmodium* viability through exposure of the parasite to low concentrations of a few selected endogenous self-aggregating peptides does not exclude the possibility that a deleterious effect on the parasite could be eventually achieved through stimulation of uncontrolled protein aggregation. Although we assume that the cytosolic localization described above can be extrapolated to most aggregative peptides delivered to *Plasmodium*, a fraction of them likely end up in other cellular subcompartments such as the digestive vacuole, thus reducing their effective cytosolic concentration and their potential stimulatory effect of protein aggregation. At this point, we decided to explore in parallel the alternative hypothesis that protein aggregation might have a functional role for *Plasmodium*. With that aim, we characterized in *P. falciparum *in vitro cultures the effect of a recently described family of β-sheet blockers that behaved as protein aggregation pan-inhibitors [40]. Interestingly, some of these compounds are 4-aminoquinolines, a chemical family that includes well-known antimalarial drugs like amodiaquine and chloroquine. Indeed, some of these 4-aminoquinoline derivatives had been described to possess antimalarial activity [39]. As controls, we included other known β-sheet intercalators such as ThT, Congo Red, and YAT2150 [56], the active component of the commercial protein aggregation detection reagent ProteoStat® (Fig. 3).

**Fig. 4:**
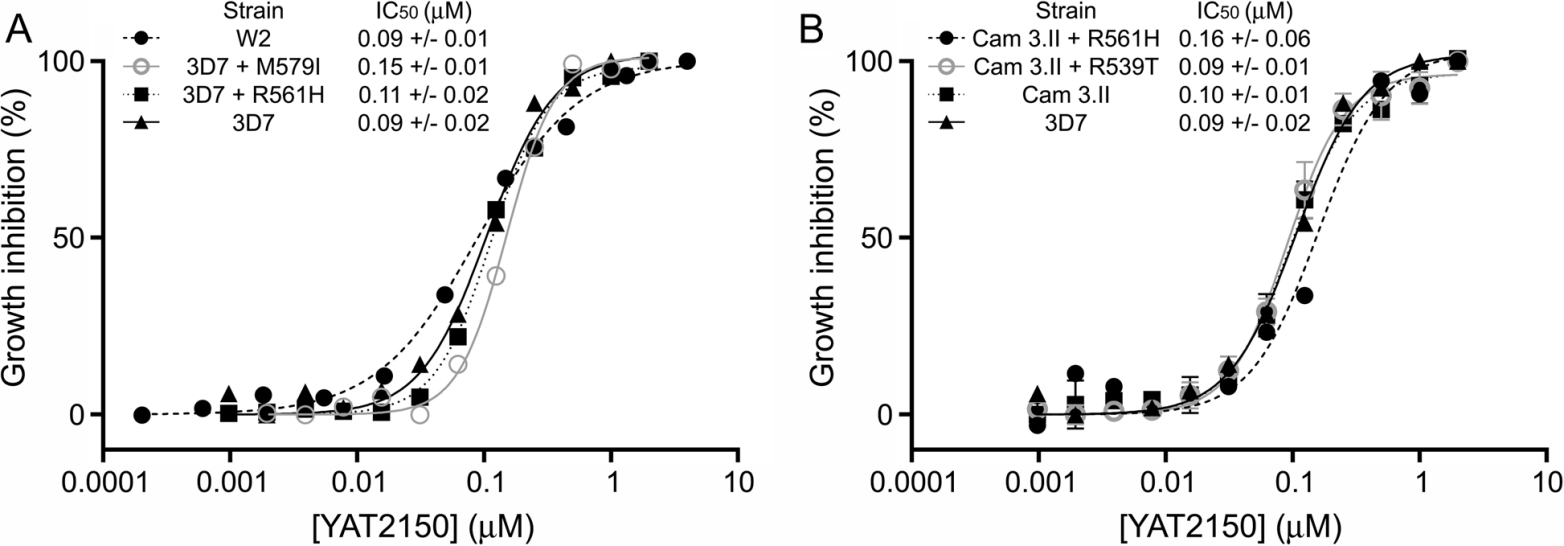
YAT2150 in vitro growth inhibition assays in chloroquine- and artemisinin-resistant strains. **A** Chloroquine-resistant W2 strain and artemisinin-resistant M579I and R561H strains compared to parental 3D7 and **B** artemisinin-resistant R561H and R539T strains compared to parental Cam 3.II and to 3D7. In both panels, means ± SD are shown

**Fig. 5:**
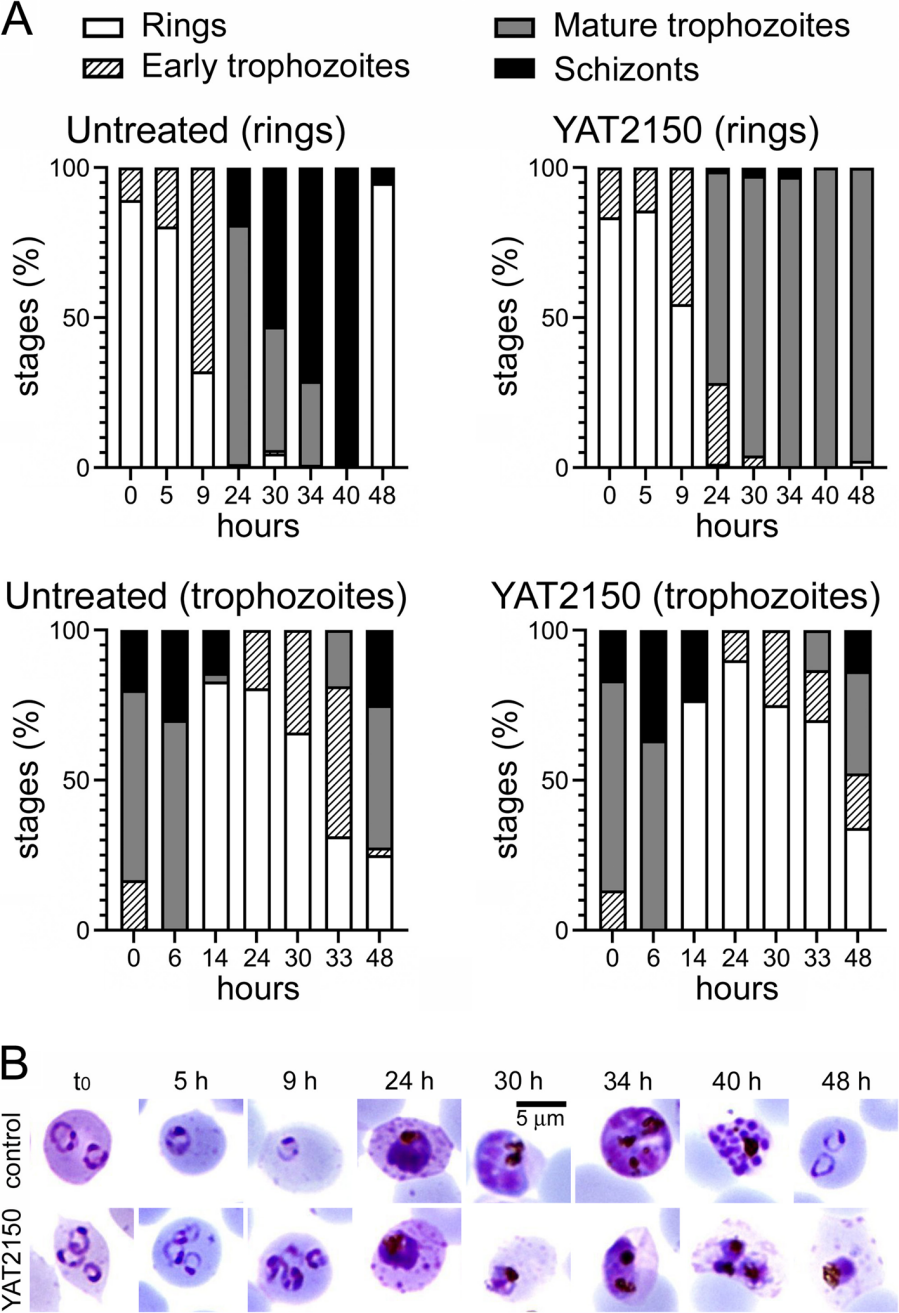
Stage of growth inhibition of *P. falciparum* during 48 h of 200 nM YAT2150 treatment. The drug was added to synchronized parasite cultures at the ring or trophozoite stages. Giemsa-stained blood smears were prepared at the indicated time points between 0 and 48 h of incubation, and the numbers of ring stages, early trophozoites, late trophozoites, and schizonts were counted in samples of at least 100 pRBCs for each time point. **A** Bars indicate the percentages of developmental stages present in the respective blood smears. **B** Representative images of pRBCs in the assay where YAT2150 was added at ring stage

The results obtained indicated that, in vitro, YAT2150 had a potent antiplasmodial activity with an IC_50_ of 90 ± 2 nM, comparable and even superior to that of most aminoquinoline amyloid pan-inhibitors previously described (Table 2). This compound, a bis(styrylpyridinium) salt, belongs to a chemical family where no antimalarial drugs have been described so far, which motivated us to characterize its activity in deeper detail. YAT2150 was also strongly active against the chloroquine-resistant W2 strain (IC_50_ of 90 ± 1 nM) and several artemisinin-resistant strains (Fig. 4), with IC_50_ values ranging from 90 to 160 nM. When added to ring stages at its IC_80_, YAT2150 arrested the life cycle of the pathogen at trophozoite stage (Fig. 5), whereas when the drug was delivered to cultures containing late *Plasmodium* forms, the parasites were able to complete their intraerythrocytic maturation and egress the pRBC, although their growth inside the new invaded RBCs became arrested at early trophozoite stage. In human umbilical vein endothelial cell cultures, the YAT2150 concentration required for the reduction of cell viability by 50% (CC_50_) was determined to be 3.4 µM (Additional file 1: Table S9), which resulted in a selectivity index (CC_50_/IC_50_) of 37.8. In vivo, YAT2150 started inducing adverse effects in female and male mice at 10 and 17 mg/kg respectively.

**Table 2:** In vitro antiplasmodial activity in *P. falciparum* cultures of amyloid pan-inhibitors and β-sheet intercalators

Compound	Chemical family	IC_50_ in 3D7 strain (µM) ± SEM	IC_50_ in K1 strain (µM) ± SEM	Selectivity index in vitro^1^
HUP5ANTRA^a^	Aminoquinoline	0.78 ± 1.86	N/D	97.7
HUP7ANTRA^a^	Aminoquinoline	7.53 ± 0.84	N/D	3.9
HUP10ANTRA^a^	Aminoquinoline	8.73 ± 1.61	N/D	9.7
HUP7TH	Aminoquinoline	0.13 ± 0.01	0.47 ± 0.36^b^	60.0
HUP8TH	Aminoquinoline	0.21 ± 0.01	0.39 ± 0.14^b^	23.3
HUP9TH	Aminoquinoline	0.16 ± 0.02	0.35 ± 0.06^b^	30.6
HUPH10TH	Aminoquinoline	0.18 ± 0.05	3.50 ± 2.29^b^	18.9
HUPNTH	Aminoquinoline	0.31 ± 0.02	0.43 ± 0.22^b^	11.0
HUPNTCl	Aminoquinoline	0.15 ± 0.01	0.52 ± 0.13^b^	42.0
DP128^c^	Aminoquinoline	0.65 ± 0.14	N/D	75.4
DONE3TCl	Aminoquinoline	0.08 ± 0.03	0.36 ± 0.07^c^	157.5
YAT2150	Bis(styrylpyridinium) salt	0.09 ± 0.02	N/D	37.8
ThT	Benzothiazolium salt	1.57 ± 0.23	N/D	N/D
Congo Red	Bis(naphthalenesulfonate)	> 15	N/D	N/D

^1^50% cytotoxic concentration (CC_50_)/IC_50_ in 3D7 strain. See Additional file 1: Table S9 for CC_50_ values

^a^[57]

^b^[39]

^c^[40]

*N/D* no data

**Fig. 6:**
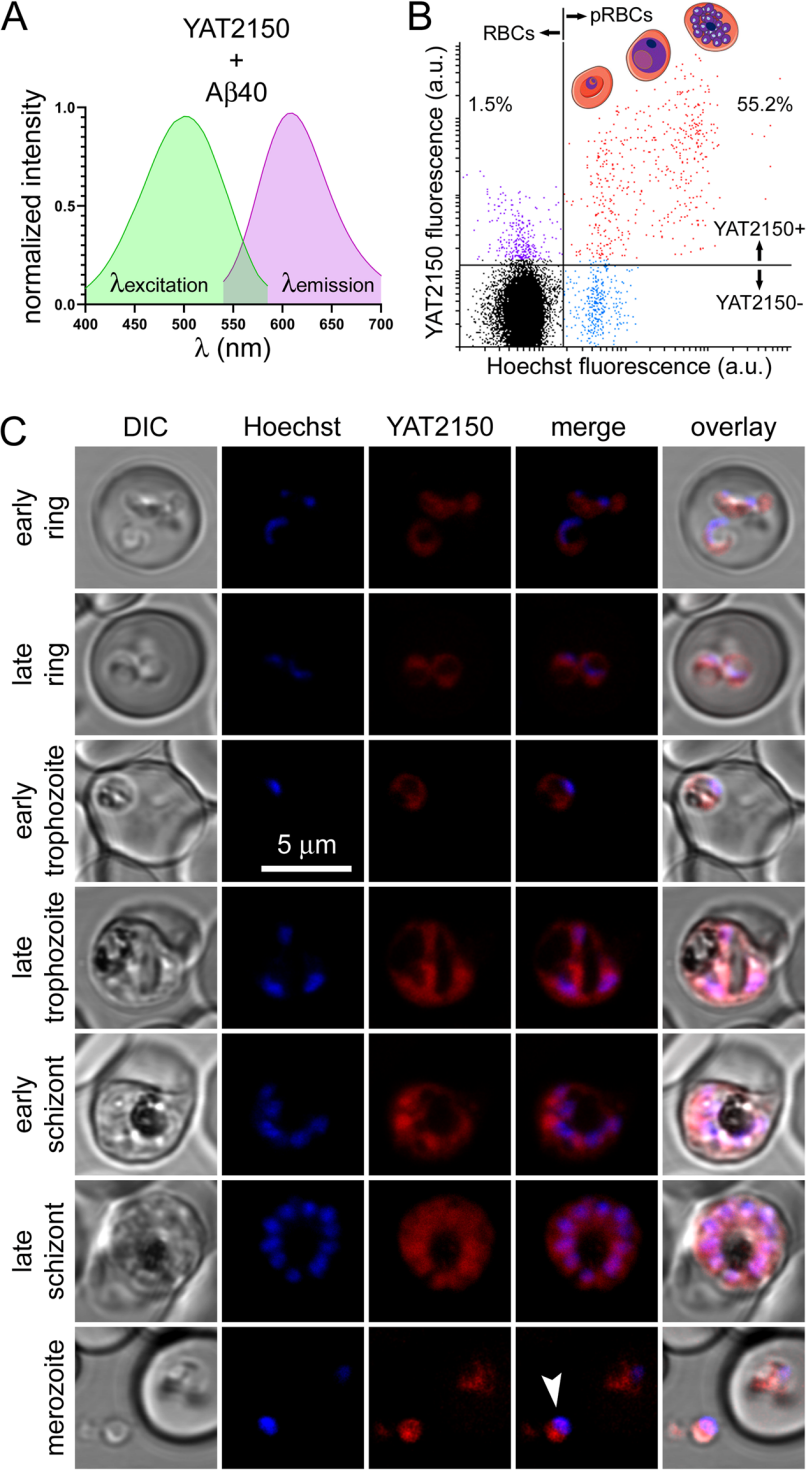
Cellular and subcellular targeting of YAT2150. **A** Absorbance and emission spectra of YAT2150 in the presence of Aβ40. **B** Flow cytometry analysis of a YAT2150-stained desynchronized *P. falciparum* culture where 30,000 events were recorded. The fraction of YAT2150-positive RBCs and pRBCs is indicated (%), the latter consisting of late rings, trophozoites and schizonts, the three stages represented in the cartoons. **C** Confocal fluorescence microscopy examination of *P. falciparum* blood stages. The arrowhead indicates an individual merozoite. DIC: differential interference contrast image

**Fig. 7:**
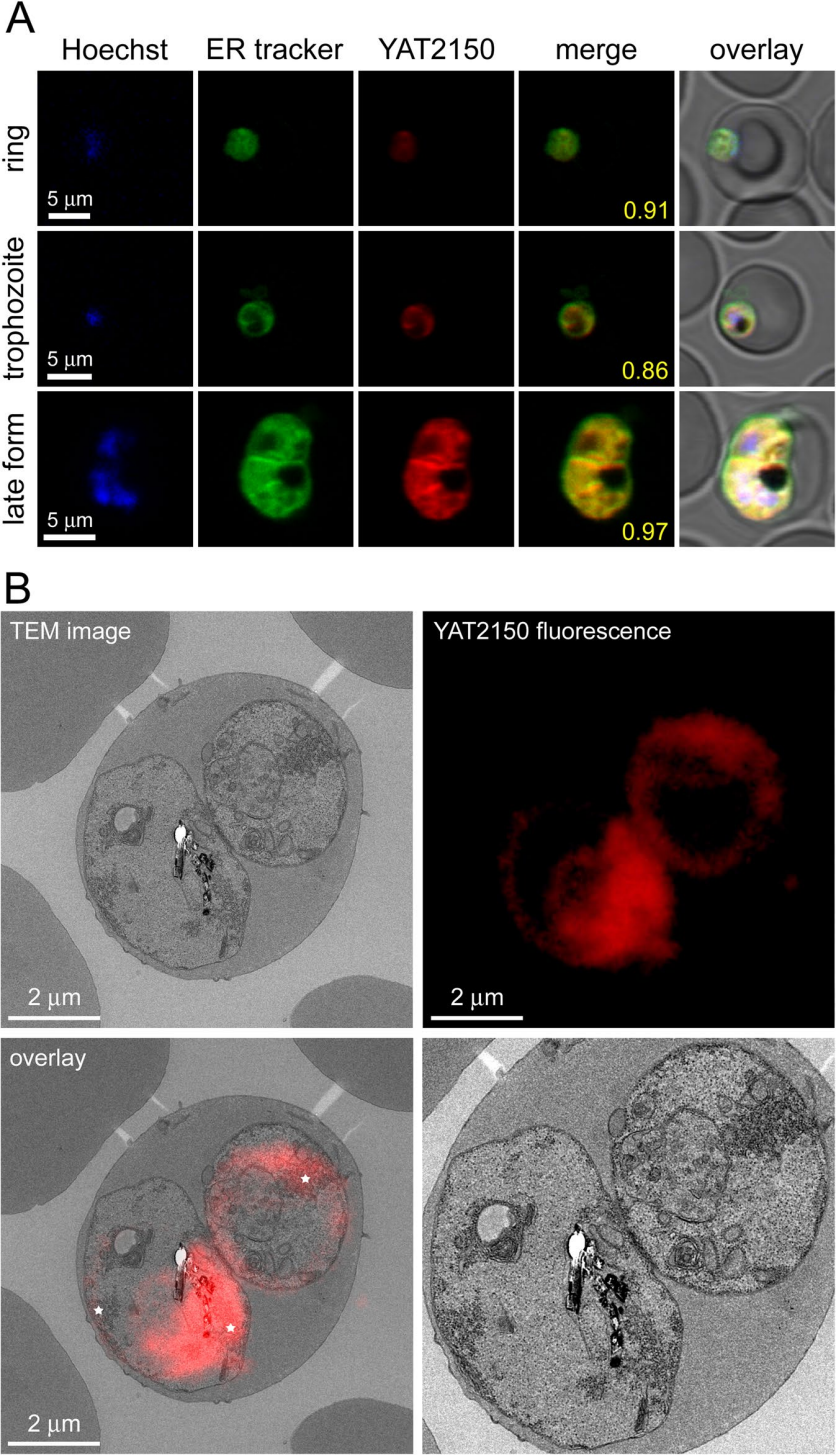
Subcellular localization of YAT2150 in pRBCs. **A** Confocal fluorescence microscopy colocalization analysis in different *P. falciparum* blood stages of YAT2150 with the cytosolic marker ER Tracker™ Green. The merge images correspond to red (YAT2150) and green (ER Tracker) channels only. Manders’ overlap correlation coefficients are indicated in yellow digits. **B** Correlative light and electron microscopy analysis. The stars in the overlay image indicate three ER regions. The blown up micrograph in the lower right panel is included for a better identification of subcellular structures

Upon binding protein aggregates (e.g., those formed by the model amyloidogenic peptide Aβ fragment 1–40, Aβ40), YAT2150 is a fluorescent molecule with respective absorbance and emission maxima at 500 and 610 nm (Fig. 6A). The fluorescence properties of YAT2150 allowed for a straightforward flow cytometry analysis of its targeting to erythrocytes (Fig. 6B), which revealed a remarkable specific targeting to pRBCs vs. non-parasitized RBCs in all blood stages, although in a significant proportion of early ring forms the fluorescent signal was below the set cytometer threshold. Confocal fluorescence microscopy imaging confirmed the presence of YAT2150 staining in all blood stages of *P. falciparum *in vitro cultures (Fig. 6C). pRBC fluorescence increased along the intraerythrocytic cycle of the parasite, with individual merozoites being strongly stained. The subcellular location of the signal was always observed inside the parasitophorous vacuole and not in the RBC cytosol. Confocal fluorescence microscopy colocalization analysis (Fig. 7A) and correlative light and electron microscopy data (Fig. 7B) confirmed the presence of YAT2150 mainly in the parasite’s cytosol, particularly in association with endoplasmic reticulum (ER) regions.

### Effect of YAT2150 on protein aggregation

**Fig. 8:**
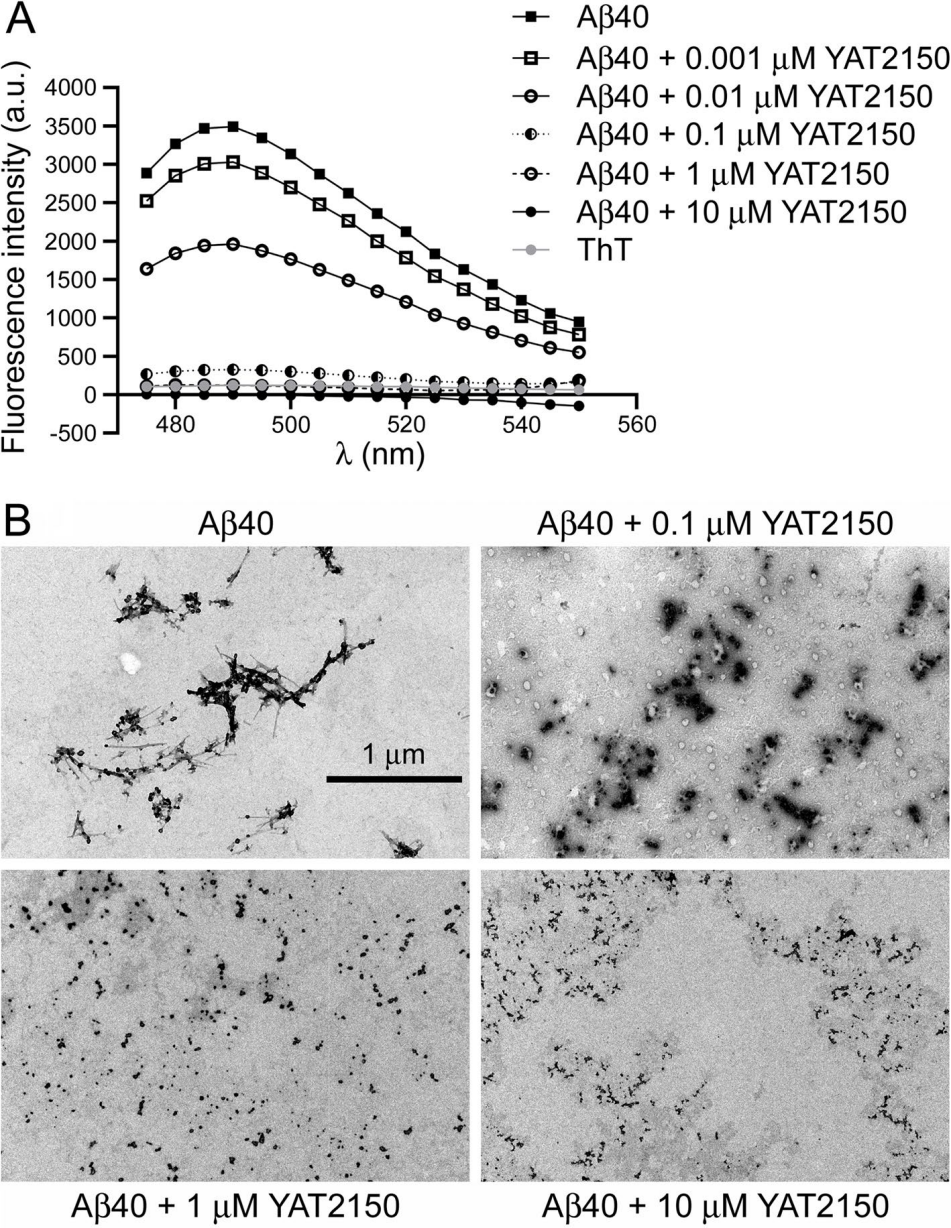
Effect of YAT2150 on the in vitro aggregation of Aβ40. **A** ThT fluorescence assay. The mean fluorescence intensity value of each sample in each wavelength is represented. a.u.: arbitrary units. **B** TEM analysis

According to in vitro ThT fluorescence assays, 0.1 µM YAT2150 is a strong inhibitor of the aggregation of Aβ40, and even at 10 nM, well below its IC_50_ in *P. falciparum* cultures, YAT2150 prevented Aβ40 fibrillogenesis to a large extent (Fig. 8A). This is in agreement with the hypothesis that inhibition of protein aggregation might be the main mechanism responsible for the antimalarial activity of this compound. To discard the possibility that the decrease in ThT fluorescence observed in Aβ40 aggregation assays resulted from a steric hindrance imposed to ThT binding of amyloid fibrils by the presence of YAT2150, we used TEM to examine Aβ40 samples treated with YAT2150 (Fig. 8B). TEM images showed that the amyloid fibril aggregates of YAT2150-containing samples were smaller and more fragmented than those present in control untreated Aβ40, supporting the role of this compound as an amyloid aggregation inhibitor. YAT2150 at concentrations > 90 nM was also found to disassemble preformed Aβ40 fibrils (Additional file 1: Fig. S9). Both in vitro aggregation inhibition and disaggregation assays show the presence of characteristic amorphous aggregates at 0.1 µM YAT2150, which might represent an intermediate species between mature Aβ40 fibrils and the disassembled protofibrillar structures found at higher drug concentrations. These aggregation inhibition and disaggregation activities were also observed with the six aggregative peptides from Fig. 1 present in *P. falciparum* proteins (Additional file 1: Fig. S10).

**Fig. 9:**
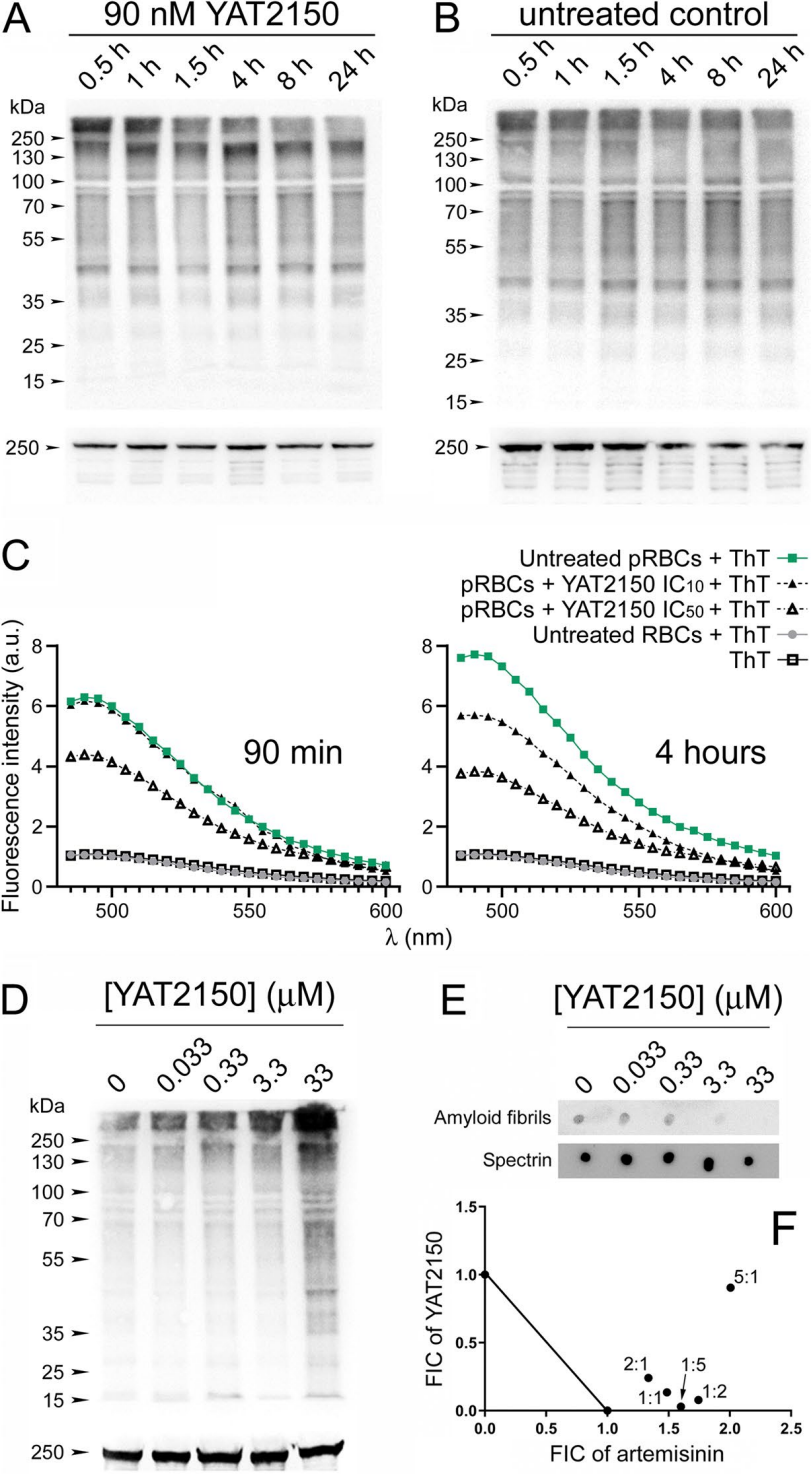
Analysis of the effect of YAT2150 on markers of protein aggregation in live *P. falciparum* cultures. **A**,**B** Western blot assays for the detection of ubiquitinated proteins in cultures treated for 0.5 to 24 h with 90 nM YAT2150. Panel B corresponds to an untreated control. Anti-spectrin antibody is used as loading control shown at the bottom of each gel. **C** ThT fluorescence of *P. falciparum* culture extracts normalized to have equal protein content, either non-treated or treated with YAT2150 at its in vitro IC_10_ (27 nM) and IC_50_ (90 nM), for 90 min and 4 h. A non-parasitized RBC protein extract is shown as reference. The mean fluorescence intensity value of each sample in each wavelength is represented. **D** Western blot assays for the detection of ubiquitinated proteins in cultures treated for 90 min with different concentrations of YAT2150. **E** Dot blot assay of the same samples from panel **D** using an antibody against amyloid fibrils. **F** Isobologram of the interaction between YAT2150 (Y) and artemisinin (A) at different Y:A ratios

Once the in vitro activity of YAT2150 as inhibitor of the aggregation of a model amyloidogenic peptide like Aβ40 and of aggregative peptides present in *P. falciparum* proteins was confirmed, we investigated if this effect could also be occurring in live parasites. To explore if there was a correlation between the observed in vitro antimalarial activity of YAT2150 and a disruption of protein homeostasis in the treated parasites, we performed Western blots and dot blot assays using protein extracts of YAT2150-treated *P. falciparum* cultures where the presence of ubiquitinated proteins and amyloid fibrils was examined (Fig. 9). Under physiological conditions, the predominant route for misfolded and aggregated protein clearance involves ubiquitination and proteasome-mediated degradation [58]. At the concentration of 90 nM, its IC_50_ in vitro, YAT2150 treatment of *P. falciparum* cultures led to a reduction along time in the fraction of ubiquitinated proteins above 250 kDa (Fig. 9A), which is enriched in protein aggregates, in agreement with an inhibitory effect on protein aggregation.

To directly probe the level of protein aggregation in live *Plasmodium* cells following YAT2150 treatment, we developed a ThT-based method to measure protein aggregation in parasite cultures. ThT fluorescence of culture extracts normalized to have equal protein content, exhibited a reduced emission spectrum in samples that had been treated for only 90 min with 90 nM YAT2150, the compound’s in vitro IC_50_ (Fig. 9C). After 4 h of treatment, the decrease in ThT fluorescence was more evident, even for cultures treated with the IC_10_ of YAT2150 (27 nM). This reduced signal could still be clearly detected after 30 h of 90 nM treatment (Additional file 1: Fig. S11). These results indicating a relevant decrease in aggregated protein load in live parasites following YAT2150 treatment at physiologically relevant concentrations are supportive of a mode of action of this drug consisting on the inhibition of protein aggregation in the pathogen.

According to dot blots using an amyloid structure-specific antibody, exposure of parasites for 90 min to > 3 µM YAT2150 inhibited amyloid fibril formation (Fig. 9E), but resulted in a concomitant increase in ubiquitinated proteins (Fig. 9D). These results suggested a causal effect between decreasing protein aggregation and a deleterious effect on the parasite ultimately leading to a rapid generalized deregulation of proteostasis.

Artemisinin, one of the most potent antimalarials in use, has been described to cause protein damage/unfolding and to inhibit folding of newly synthesized proteins, likely inducing protein aggregation [25]. When *P. falciparum* cultures were treated with artemisinin and YAT2150 combined at different ratios, the resulting fractional inhibitory concentration values were always higher than 1.5 (Fig. 9F), which indicated an antagonistic action of both drugs [59]. Thus, parasite viability was higher than expected for drug synergism, supporting the hypothesis that YAT2150 has protein aggregation inhibitory activity that antagonizes the antimalarial effect of artemisinin, and vice versa. In Fig. 4, we tested the effect of YAT2150 in 3D7 *P. falciparum* parasites harboring the K13 mutations associated to artemisinin resistance M579I and R561H [60], and in the multiresistant Cam 3.II strain, which, besides being resistant to chloroquine and sulfadoxine/pyrimethamine, it had been modified to carry the K13 mutations R561H and R539T [60]. The results did not show a significant IC_50_ increase relative to the corresponding parental strains except for M579I (*p*-value of 0.02; all other *p*-values > 0.15), in agreement with a mode of action for YAT2150 different from that of artemisinin.

YAT2150 did not block the formation of hemozoin (Additional file 1: Fig. S12), thus confirming that its antimalarial mechanism is different from that of the widely used quinoline drugs like chloroquine. This result is in agreement with the calculated IC_50_ of YAT2150 in the chloroquine-resistant *P. falciparum* W2 strain (Fig. 4A), undistinguishable from its activity in the chloroquine-sensitive 3D7 strain.

### Activity of YAT2150 on *Plasmodium* gametocytes and liver stages

**Fig. 10:**
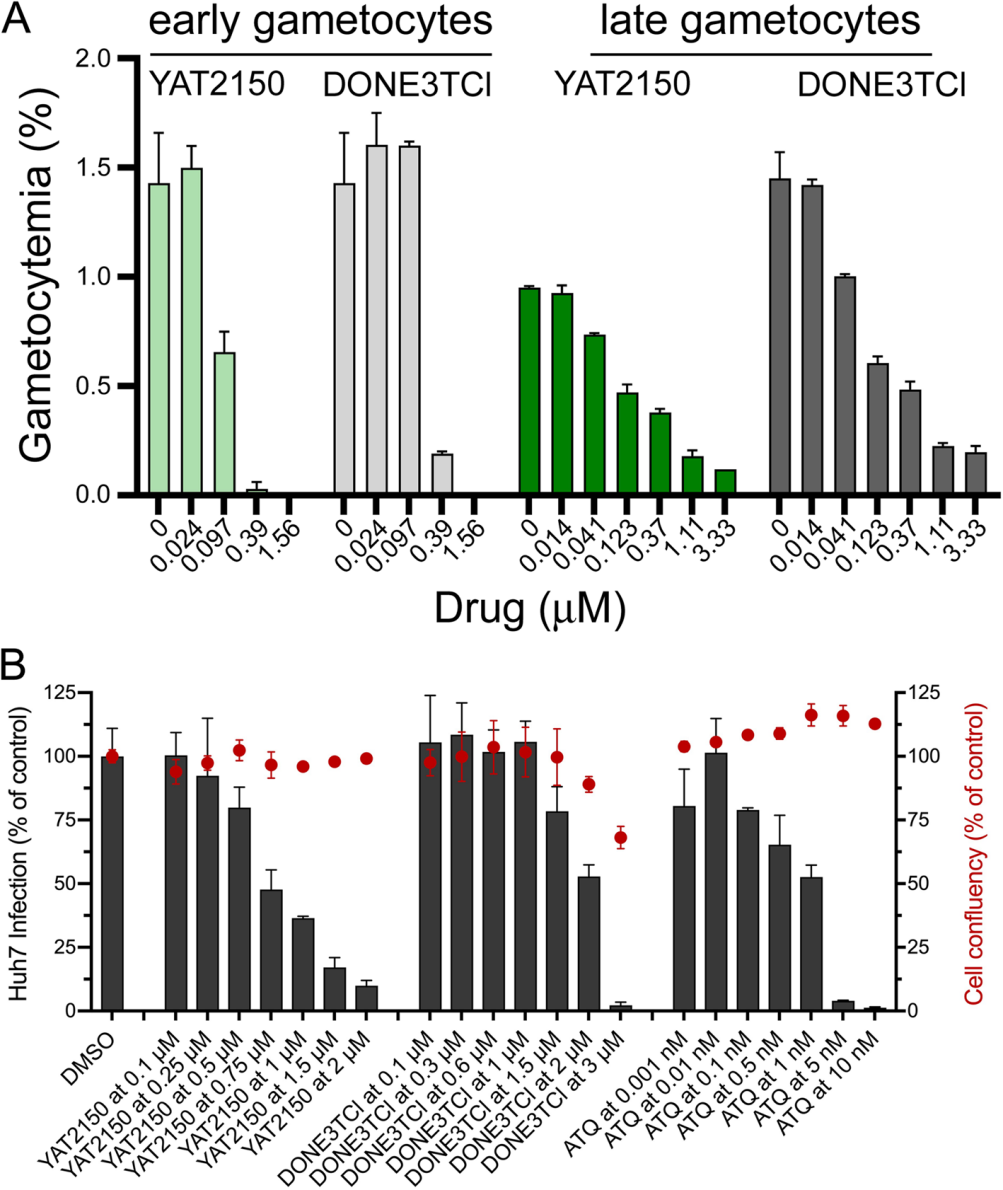
In vitro activity of YAT2150 and DONE3TCl on *Plasmodium* gametocytes and liver stages. **A** Effect of the drugs on *P. falciparum* early and late gametocytes. Bars represent mean ± SD of two independent experiments. **B** Dose-dependent response of YAT2150, DONE3TCl, and atovaquone (ATQ) against the hepatic stage of *P. berghei* infection. Total parasite load (infection scale, bars) and cell viability (cell confluency scale, red dots) are shown. Results were normalized to DMSO and are represented as mean ± SD of three independent experiments. Reference drug is atovaquone (IC_50_ = 1.63 ± 0.27 nM)

The recent appreciation that efficient antimalarial strategies will require the interruption of parasite transmission from the human host to the vector [61] has prompted the search for transmission-blocking drugs [62]. Targeting gametocytes, the sole stage of malaria parasites present in the blood circulation capable of transmitting the infection to the mosquito vector following their ingestion by a blood-feeding *Anopheles* female, can ease exposure of the pathogen to drugs and reduce the likelihood of the emergence of resistance [63]. However, although eliminating gametocytes is one of the main approaches being explored to disrupt the life cycle of *Plasmodium*, drugs active at this critical step are scarce [64]. YAT2150 efficiently blocked the development of *P. falciparum* early and mature stage V gametocytes in vitro with respective IC_50_ of 95 ± 3 nM and 103 ± 3 nM (Fig. 10A), close to that obtained for the asexual blood stages. For the amyloid pan-inhibitor aminoquinoline DONE3TCl, the IC_50_ values on early and late gametocytes were 285 ± 56 nM and 78 ± 12 nM, respectively.

Finally, targeting the liver stage of malaria parasites is a key therapeutic and prophylactic antimalarial strategy because its blockade would impair the subsequent RBC invasion, thus preventing progression to symptomatic disease. Therefore, we assessed the in vitro activity of YAT2150 against hepatic infection by *Plasmodium.* To this end, we employed a well-established infection platform based on the use of luciferase-expressing rodent malaria *Plasmodium berghei* parasites and the human hepatoma cell line Huh7. Our results showed that YAT2150 significantly inhibited the infection of hepatic cells by *P. berghei*, with an estimated IC_50_ of 0.78 µM (Fig. 10B). DONE3TCl, used as a control in this experiment, revealed only modest activity at 1.5 µM and was toxic against the Huh7 host cells at concentrations ≥ 2 µM (Fig. 10B), confirming the specificity of YAT2150’s hepatic stage antiplasmodial activity.

## Discussion

Perhaps the main threat to malaria elimination and its eventual eradication is the evolution by the parasite of resistance to every drug deployed against it [65]. Drug resistance arises with the spontaneous emergence of mutations or gene duplications conferring reduced drug susceptibility, which are then selected by the presence of low local drug concentrations insufficient to suppress the growth of resistant clones. Although novel regimens and strategies for the better use of existing antimalarial drugs are required, the deployment of novel compounds is an urgent need, given the paucity of their appearance in the clinical arena. Among the most desirable properties of future antimalarials in order to minimize the risk of resistance evolution are (i) a low IC_50_ to allow for the safe administration of lethal doses to the parasite, (ii) classification into a chemical group where no antimalarials have been described so far to minimize the risk of adaptation of existing resistance mechanisms, and (iii) a target present in several stages of the pathogen and which is not a single-gene product to reduce the chances that resistance can appear rapidly.

The potential toxicity for the cell of protein aggregates, the high amount of aggregation-prone proteins in *Plasmodium*, and the specificity of seeding in protein aggregation reactions suggested that, a priori, the specific exacerbation of the propensity of the parasite’s proteome to aggregate could be exploited for therapeutic purposes. However, aggregative peptides present in *P. falciparum* proteins were not observed to have significant toxicity for the parasite when exogenously incorporated to in vitro cultures at clinically relevant concentrations. This result indicates that the targeting of a few gene products where the peptides are present does not suffice to significantly impair *Plasmodium* viability, although an effective prevention of aggregation by the parasite chaperones cannot be ruled out. Actually, the cytoplasmic *P. falciparum* heat shock protein 110 (*Pf*Hsp110c) has been proved to be 15 to 30 times better than its yeast or human orthologs at preventing aggregation of Q/N repeat-enriched proteins in mammalian cells [66]. However, disruption in *P. falciparum* of the ubiquitin-dependent protein disposal pathway through inhibition of ubiquitin E3 ligase has been proposed as an antimalarial strategy [67]. The same enzyme has been identified here as a protein with a high propensity to aggregate and therefore a potential target for antiplasmodial approaches based on overloading the parasite’s proteasome. Further research is required to explore this possibility in deeper detail.

On the other hand, the use of pan-inhibitors of protein aggregation leads to a disruption of proteostasis by affecting multiple gene products, and it is therefore unlikely to elicit rapid evolution of resistance. This alternative strategy did dramatically reduce the viability of the malaria parasite. Several compounds with diverse chemotypes that inhibit protein aggregation have been shown to possess antimalarial activity in in vitro* P. falciparum* cultures. In this scenario, evolution of resistance by the pathogen would be further complicated by the need to evolve different ad hoc resistance mechanisms for each individual drug if these were used in combination. For those molecules belonging to chemical families where currently used antimalarials belong, resistance might be achieved with relative rapidity through the adaptation of already existing resistance mechanisms. As an example of this, resistance to the aminoquinolines chloroquine and piperaquine has been associated with distinct sets of point mutations in the *P. falciparum* chloroquine resistance transporter *Pf*CRT, an efflux pump evolved by the parasite to expel chloroquine [68]. However, resistance would be slowed down significantly for those molecules belonging to chemical families where no antimalarials have been described so far, such as YAT2150, a double styrylpyridinium salt with an IC_50_ in *P. falciparum* cultures of ca. 90 nM. The sensitivity to YAT2150 of the chloroquine-resistant W2 strain and the results of hemozoin formation assays indeed indicate that the antimalarial mode of action of YAT2150 is not related to that of chloroquine.

YAT2150 specifically targets pRBCs vs. non-parasitized RBCs and binds aggregates in all *Plasmodium* stages, including early blood forms, and therefore its inhibitory effect on protein aggregation can be manifested at the start of the intraerythrocytic cycle, thus maximizing its antimalarial activity. *P. falciparum* overall ubiquitination increases as parasites mature from ring through trophozoite and schizont to merozoite forms [69], mirroring the observed increase in YAT2150 fluorescence along the blood stage cycle, in agreement with a scenario where protein aggregation mounts to reach its maximum with merozoite egress. The reduced signal observed in a significant fraction of early ring stages suggests that protein aggregates are required for merozoite invasion of the RBC but are lost in the process, which would set the aggregation clock of the pathogen back to zero at the moment of parasitizing a new erythrocyte.

Besides its activity against *Plasmodium* asexual blood stages, YAT2150 displays marked activity against the sexual and hepatic phases of the malaria parasites’ life cycle, paving the way for the exploration of this drug as a potential multi-stage antiplasmodial therapy. Other attractive characteristics of this compound are an easy, rapid, and inexpensive synthesis, long room temperature storage, and its fluorescent emission when binding protein aggregates in the pathogen, which offers an added malaria diagnosis potential. YAT2150 can then be defined as a malaria theranostic agent.

Artemisinin, which in live parasites induces protein aggregation [24, 25], has been described to inhibit in vitro the aggregation of amyloid peptides [70, 71]. However, our data showing a sensitivity of artemisinin-resistant strains to YAT2150 similar to that of the corresponding parental non-resistant lines, strongly suggests that the antimalarial modes of action of both drugs are not related. Actually, in *P. falciparum* cultures, we have observed an antagonistic action of artemisinin and YAT2150, indicating that their main effects on the pathogen are opposed and therefore their combined use should a priori not be recommended. However, by 2016 the emergence of artemisinin and partner drug resistance in *P. falciparum* was detected in the Greater Mekong Subregion [72], and recently, the independent evolution of artemisinin resistance has also been reported in Africa [73] and South America [74]. This alarming scenario calls for the urgent development of new drugs like YAT2150 with little-exploited targets in the malaria parasite.

The heme group released from hemoglobin as *Plasmodium* feeds on it has also been shown to promote the aggregation of proteins in the cell [75, 76], which could explain the reduced presence of protein aggregates in early ring stages of the parasite. The potent blocking of the aggregation of Aβ40 and of aggregative peptides present in parasite proteins by YAT2150 at concentrations inhibiting parasite growth in in vitro cultures suggests that this drug initially causes in live parasites inhibition of protein aggregation, which is presumably functional for *Plasmodium*. This hypothesis is supported by the observed inhibition of protein aggregation in *P. falciparum* cultures at the compound’s physiologically relevant IC_50_. At the same concentration, YAT2150 disassembles in vitro preformed aggregates/fibrils of Aβ40 and of aggregative peptides found in *P. falciparum* proteins, in agreement with the existence of functional protein aggregates that are required for parasite survival. The cytosolic localization of the protein aggregation inhibitor YAT2150 in *Plasmodium* rough ER regions, where proteins are being synthesized, is consistent with the likely role of this drug in disrupting a yet to be described parasite’s aggresome.

Our data obtained at high YAT2150 concentrations show a reduction of amyloid fibril content, which is mirrored by a simultaneous massive increase in ubiquitinated proteins, suggesting the existence in *Plasmodium* of functional amyloidogenic protein regions that, if disrupted by amyloid inhibitors, eventually assemble into amorphous aggregates. We propose that this interference with the as yet unknown role of certain aggregative proteins eventually triggers adverse physiological alterations in the parasite ultimately leading to a generalized deregulation of proteostasis. This scenario would conciliate the apparently contradictory observations regarding the effect of some antimalarials on protein aggregation in the pathogen.

## Conclusion

The data presented above suggest that further increasing protein aggregation in the already aggregate-overloaded *Plasmodium* cell does not significantly affect the viability of the parasite, whereas aggregation inhibition has clear deleterious effects for it. This finding strongly suggests that certain functional protein aggregates can be crucial for the survival of malaria parasites and for the progression of their pathological effects. Because some of these presumably functional protein assemblies might have an amyloid nature according to the results obtained with endogenous aggregative peptides identified in the pathogen’s proteome, the description of malaria as an amyloidosis should probably be considered. This might spur the search for new antiplasmodials whose mode of action is the inhibition of protein aggregation in the parasite, such as YAT2150, whose promising characteristics can make it the spearhead of a new generation of antimalarial drugs for the post-artemisinin era.

## Methods

Except where otherwise indicated, reagents were purchased from Sigma-Aldrich Corporation (St. Louis, MO, US), and reactions were performed at room temperature (RT; 22 to 24 °C). Peptides labeled on their N-terminal ends with 5(6)-carboxyfluorescein succinimidyl ester mixed isomers (5/6-FAM) were purchased from CASLO ApS, c/o Technical University of Denmark (Kongens Lyngby, Denmark), or synthesized in-house (see below). All the peptides used in this work were amidated in their C-terminal ends. Aggregation-prone sequences fused to cell-penetrating peptide motifs were prepared as described below. Except otherwise stated in the figure and table legends, all assays were replicated in at least three independent experiments maintaining the same experimental conditions. The most representative biological replicate is shown.

### Synthesis of peptides elongated with cell-penetrating motifs

*P. falciparum* aggregation-prone peptides linked to the CPPs LMWP (VSRRRRRRGGRRRR) [77], TAT (GRKKRRQRRRPPQ) [51], or TP2 (PLIYLRLLRGQF) [78] were produced by solid-phase synthesis in Prelude (Gyros Protein Technologies, Tucson, AZ, US) or Liberty Blue instruments (CEM, Matthews, NC, US). Fivefold excess of fluorenylmethoxycarbonyl (Fmoc)-amino acids dissolved in *N*,*N*-dimethylformamide (DMF) were coupled in the presence of 2-(1*H*-benzotriazol-1-yl)-1,1,3,3-tetramethyluronium hexafluorophosphate (fivefold molar excess) and *N*,*N*-diisopropylethylamine (tenfold molar excess). After coupling and washing with DMF, Fmoc removal was done with 20% piperidine in DMF. Upon completion of the synthesis, the peptide resin was deprotected as described above, washed with dichloromethane and DMF, and, if required, reacted with 5/6-FAM activated with *N*,*N'*-diisopropylcarbodiimide (tenfold molar excess of both reagents). Then the peptides were side-chain deprotected and cleaved from the resin with 95% (v/v) trifluoroacetic acid (TFA), 2.5% (v/v) triisopropylsilane, and 2.5% (v/v) water. Two-hundred milligrams of each peptide resin was treated with 5 ml of cleavage cocktail for 2 h at RT. Resin was removed by filtration and peptides in TFA solution were isolated by precipitation with cold diethyl ether and centrifugation (2 × 10 min at 2000 × *g*); supernatant was removed and the peptide pellet was dried. Next, the crude peptide was taken up in water for high-performance liquid chromatography (HPLC) and mass spectrometry (MS) analyses. HPLC analysis was performed with C18 columns (4.6 × 50 mm, 3 μm; Phenomenex, Torrance, CA, US) in a Shimadzu LC-2010A liquid chromatograph (Shimadzu Corporation, Kyoto, Japan). Solvent A was 0.045% TFA in H_2_O, and solvent B was 0.036% TFA in acetonitrile. Elution was carried out with linear gradients (10–50% for LMWP- and TAT-peptides and 30–65% for TP2-peptides) of solvent B into solvent A over 15 min at 1 ml/min flow rate, with UV detection at 220 nm. MS was performed in a LC–MS 2010EV instrument (Shimadzu Corporation) fitted with an XBridge column (4.6 × 150 mm, 3.5 μm; Waters Corporation, Milford, MA, US). Peptides were eluted with the same linear gradients used for HPLC of solvent B into solvent A (A: 0.1% formic acid in H_2_O; B: 0.08% formic acid in acetonitrile).

Preparative HPLC runs were performed on a Luna C18 column (21.2 mm × 250 mm, 10 μm; Phenomenex), using the same linear gradients as for HPLC and MS of solvent B (0.1% TFA in acetonitrile) into A (0.1% TFA in H_2_O), as required, with a flow rate of 25 ml/min. Fractions with > 95% homogeneity were further characterized by electrospray mass spectrometry using a XBridge column C18 (Waters Corporation) and a gradient at 1 ml/min of solvent A (0.1% formic acid in H_2_O) into solvent B (0.08% formic acid in acetonitrile), with 220 nm detection. Those with the expected HPLC homogeneity and mass were pooled, lyophilized, and used in subsequent experiments.

### Synthesis of YAT2150 (dibromide salt)

All reagents and solvents were obtained from commercial suppliers and used without further purification. Automatic flash column chromatography was performed on a CombiFlash Rf 150 (Teledyne Isco) with prepacked RediSep Rf silica gel cartridges. Melting points were determined in open capillary tubes with a MFB 595010 M Gallenkamp melting point apparatus. IR spectra were run on a Perkin Elmer Spectrum RX I spectrophotometer. Absorption values are expressed as wavenumbers (cm^−1^). Then, 500 MHz ^1^H / 125 MHz ^13^C NMR spectra were recorded on a Bruker Avance Neo 500 MHz spectrometer, at the *Centres Científics i Tecnològics* of the University of Barcelona (CCiTUB). The chemical shifts are reported in ppm (*δ* scale) relative to dimethyl sulfoxide (DMSO) solvent signals (DMSO-d_6_ at 2.50 and 39.5 ppm in the ^1^H and ^13^C NMR spectra, respectively), and coupling constants are reported in Hertz (Hz). Assignments given for the NMR spectra have been carried out on the basis of DEPT and COSY ^1^H/^13^C (gHSQC sequences) experiments. High-resolution mass spectra were carried out at the CCiTUB with a LC/MSD TOF Agilent Technologies spectrometer.

A mixture of 1,10-dibromodecane (1.50 g, 5.00 mmol) and 3,4-dimethylpyridine (1.2 ml, 1.14 g, 10.7 mmol) was heated at 120 °C for 3 h. Then, isopropanol (5 ml) was added, and the reaction mixture was stirred under reflux for 1 h. The mixture was allowed to cool down to RT, the resulting brown residue was washed with ice-cold Et_2_O (2 × 40 ml), the supernatant was removed and the remaining brown sticky oil was dried *in vacuo*, taken up in MeOH (1 ml) and treated with cold Et_2_O (2 × 40 ml), drawing off the liquids. After drying the residue *in vacuo*, 1,1'-(decane-1,10-diyl)*bis*(3,4-dimethylpyridin-1-ium) dibromide (2.48 g, 96%) was obtained as a brown oil that solidified on standing; mp: 69–71 °C; IR (ATR) *ν*: 3443, 3396, 3027, 2988, 2921, 2851, 1635, 1512, 1483, 1471, 1391, 1224, 1143, 1031, 870, 838, 710, 597, 559 cm^−1^; ^1^H NMR (500 MHz, DMSO-d_6_) *δ*: 1.20–1.32 [m, 12H, 3’(8’)-H_2_, 4’(7’)-H_2_, 5’(6’)-H_2_], 1.89 [tt, *J* = *J’* = 7.5 Hz, 4H, 2’(9’)-H_2_], 2.40 (s, 6H, pyridinium 3-CH_3_), 2.52 (s, 6H, pyridinium 4-CH_3_), 4.50 [t, *J* = 7.5 Hz, 4H, 1’(10’)-H_2_], 7.95 (d, *J* = 6.0 Hz, 2H, pyridinium 5-H), 8.85 (dd, *J* = 6.0 Hz, *J’* = 1.5 Hz, 2H, pyridinium 6-H), 8.96 (br s, 2H, pyridinium 2-H); ^13^C NMR (125 MHz, DMSO-d_6_) *δ*: 16.3 (2 CH_3_, pyridinium 3-CH_3_), 19.6 (2 CH_3_, pyridinium 4-CH_3_), 25.4 (2 CH_2_), 28.3 (2 CH_2_), 28.7 (2 CH_2_) [C3’(8’), C4’(7’), C5’(6’)], 30.5 [2 CH_2_, C2’(9’)], 59.7 [2 CH_2_, C1’(10’)], 127.9 (2 CH, pyridinium C5), 137.6 (2 C, pyridinium C3), 141.5 (2 CH, pyridinium C6), 142.8 (2 CH, pyridinium C2), 157.6 (2 C, pyridinium C4); HRMS-ESI + *m/z* calculated for [C_24_H_38_N_2_]^2+^/2: 177.1512, found 177.1513.

A solution of 1,1'-(decane-1,10-diyl)*bis*(3,4-dimethylpyridin-1-ium) dibromide (514 mg, 1.00 mmol) and 4-(diethylamino)benzaldehyde (390 mg, 2.20 mmol) in *n*-butanol (5 ml) was treated with six drops of piperidine and the reaction mixture was stirred under reflux for 4 h, and then concentrated under reduced pressure. The resulting black oily residue was purified by automatic flash column chromatography (CH_2_Cl_2_ / 7 N ammonia solution in MeOH 9:1), to provide 1,1'-(decane-1,10-diyl)*bis*{4-[(*E*)-4-(diethylamino)styryl]-3-methylpyridin-1-ium} dibromide (351 mg, 42%) as a red oil that solidified on standing; mp: 173–174 °C; IR (ATR) *ν*: 3399, 2975, 2927, 2853, 1641, 1574, 1520, 1479, 1404, 1351, 1311, 1260, 1219, 1186, 1128, 1076, 1011, 958, 807, 572 cm^−1^; ^1^H NMR (500 MHz, DMSO-d_6_) *δ*: 1.13 [t, *J* = 7.0 Hz, 12H, N(CH_2_-C*H*_3_)_2_], 1.21–1.31 [m, 12H, 3’(8’)-H_2_, 4’(7’)-H_2_, 5’(6’)-H_2_], 1.87 [tt, *J* = *J’* = 7.5 Hz, 4H, 2’(9’)-H_2_], 2.48 (s, 6H, pyridinium 3-CH_3_), 3.43 [q, *J* = 7.0 Hz, 8H, N(C*H*_2_-CH_3_)_2_], 4.37 [t, *J* = 7.5 Hz, 4H, 1’(10’)-H_2_], 6.74 [d, *J* = 9.0 Hz, 4H, phenylene 3(5)-H], 7.08 (d, *J* = 16.0 Hz, 2H, pyridinium C4-C*H* = CH), 7.64 [d, *J* = 9.0 Hz, 4H, phenylene 2(6)-H], 7.88 (d, *J* = 16.0 Hz, 2H, pyridinium C4-CH = C*H*), 8.26 (d, *J* = 6.5 Hz, 2H, pyridinium 5-H), 8.66 (dd, *J* = 6.5 Hz, *J’* = 1.5 Hz, 2H, pyridinium 6-H), 8.73 (br s, 2H, pyridinium 2-H); ^13^C NMR (125 MHz, DMSO-d_6_) *δ*: 12.5 [4 CH_3_, N(CH_2_-*C*H_3_)_2_], 16.5 (2 CH_3_, pyridinium 3-CH_3_), 25.5 (2 CH_2_), 28.4 (2 CH_2_), 28.7 (2 CH_2_) [C3’(8’), C4’(7’), C5’(6’)], 30.5 [2 CH_2_, C2’(9’)], 43.9 [4 CH_2_, N(*C*H_2_-CH_3_)_2_], 58.9 [2 CH_2_, C1’(10’)], 111.3 [4 CH, phenylene C3(5)], 113.4 (2 CH, pyridinium C4-*C*H = CH), 119.8 (2 CH, pyridinium C5), 122.1 (2 C, phenylene C1), 130.8 [4 CH, phenylene C2(6)], 132.9 (2 C, pyridinium C3), 140.5 (2 CH, pyridinium C6), 142.4 (2 CH, pyridinium C4-CH = *C*H), 143.3 (2 CH, pyridinium C2), 149.5 (2 C, phenylene C4), 152.4 (2 C, pyridinium C4); HRMS-ESI + *m/z* calculated for [C_46_H_64_N_4_]^2+^/2: 336.2560, found 336.2550.

For its use in the assays reported below, the final product, YAT2150, was dissolved in DMSO to obtain a 9 mM stock solution.

### In vitro peptide aggregation assays

Peptide stocks prepared in DMSO were diluted in phosphate buffered saline (PBS) at a final concentration of 150 μM. After vigorous vortexing, peptides were incubated at 37 °C and 1400 rpm in a ThermoMixer® (Eppendorf, Hamburg, Germany) for 48 h. After that time, peptides were further diluted in triplicates to 25 μM in PBS, ThT was added at the same final concentration in PBS, and fluorescence emission was collected from 470 to 600 nm using an excitation wavelength of 450 nm (Infinite Nano + multimode microplate reader, Tecan Trading AG, Männedorf, Switzerland). A blank measurement of each sample was done before adding ThT.

Fluorescein-labeled peptides were diluted in PBS at 150 µM and incubated for 24 h at 37 °C and 1400 rpm. After this time, peptides were further diluted in triplicates in PBS to a final concentration of 15 µM, to which the protein aggregation detection reagent ProteoStat® (Enzo Life Sciences, Inc., Farmingdale, NY, US) was added at 1:1000 final dilution and transferred to a 96-well black plate (Greiner Bio-One, Madrid, Spain). ProteoStat® fluorescence was quantified (Tecan Infinite 200 PRO, Tecan Trading AG) using respective excitation and emission wavelengths of 550 and 600 nm. The fluorescence of a ProteoStat®-only control was also measured and subtracted from the sample values.

For the in vitro analysis of Aβ40 aggregation, 1 mg of Aβ40 (GenScript Biotech, Piscataway, NJ, US) was dissolved in 500 µl of 1,1,1,3,3,3-hexafluoro-2-propanol (HFIP; Honeywell Fluka-Thermo Fisher Scientific, Waltham, MA, US) under vigorous stirring for 1 h and sonicated for 30 min in an ultrasound bath. Afterwards, the solution was stirred for 1 h and maintained at 4 °C for 30 min. Aliquots were prepared, HFIP was evaporated under a nitrogen stream for a few seconds and the dry peptide was stored at − 20 °C. Prior to use, these Aβ40 aliquots were dissolved in DMSO and sonicated for 10 min to ensure minimal aggregation. To assess the effect of YAT2150 on the formation of amyloid fibrils, Aβ40 DMSO solutions were diluted to 25 µM in PBS containing different concentrations of YAT2150 and incubated for 24 h at 37 °C and 1400 rpm. Alternatively, to test the effect of YAT2150 on already formed amyloid fibrils, Aβ40 DMSO solutions were diluted to 25 µM in PBS and incubated as above in order to allow fibril formation. Then, YAT2150 was added at different concentrations and the mixture was incubated in the same conditions for another 24 h. The final samples always contained less than 5% DMSO to avoid interference of this solvent on Aβ40 amyloid fibril formation. Finally, ThT treatment was performed as described above. The analyses of aggregation inhibition and disaggregation performed with aggregative peptides present in *P. falciparum* proteins were conducted in the same way.

### *P*.* falciparum* growth inhibition assays

*P. falciparum* parasites of the 3D7 (MRA-102, chloroquine-sensitive) and W2 (MRA-157, chloroquine-resistant) strains (both from Malaria Research and Reference Reagent Resource Center, Manassas, VA, US), and Cam 3.II (chloroquine and sulfadoxine/pyrimethamine resistance), Cam 3.II + K13 R561H, Cam 3.II + K13 R539T, 3D7 + K13 R561H, and 3D7 + K13 M579I strains (all carrying artemisinin resistance, developed and authenticated by Stokes et al. [60] and kindly donated by Prof. David A. Fidock) were 5% sorbitol-synchronized as described elsewhere [79] in order to obtain a culture enriched in ring stage parasites. After the synchronization process, a new culture at 1.5% parasitemia and 2% hematocrit was established and 150-µl aliquots of it were transferred to 96-well plates. The required amounts of peptides, antimalarial drugs, or amyloid pan-inhibitors were added to each well at different concentrations and in triplicates. For synergy assays of YAT2150 and artemisinin, serial dilutions of both compounds were prepared at different concentration ratios (1:0, 0:1, 1:1, 1:2, 2:1, 1:5, and 5:1) as explained elsewhere [80]. A positive growth control of untreated parasites and a negative growth control of parasites treated with a lethal dose of chloroquine (1 µM) were also included. Parasites were grown for 48 h, a complete replication cycle, in standard culturing conditions (5% O_2_, 5% CO_2_, and 90% N_2_ at 37 °C). After the incubation period, 3 µl of culture from each well were mixed with 197 µl of PBS containing 0.1 µM Syto 11 (Thermo Fisher Scientific), to obtain a final concentration of ca. 1–10 × 10^6^ cells/ml. Parasitemia was assessed by flow cytometry using a LSRFortessa flow cytometer (BD Biosciences, San Jose, CA, US) set up with the 4 lasers, 20 parameters standard configuration. The single-cell population was selected on a forward-side scattergram. Syto 11 fluorescence signal was detected by exciting samples at 488 nm and collecting the emission with a 530/30-nm bandpass filter. Growth inhibition was calculated taking as reference values both the growth rate of the untreated culture and the growth rate of the culture treated with chloroquine. Growth inhibition data was transformed through sigmoidal fitting and used to determine the compound’s concentration required for the reduction of *P. falciparum* viability by 50% (IC_50_).

To assess the synergistic effect of YAT2150 and artemisinin, IC_50_ values for each individual compound in the mixtures were calculated and plotted in an isobologram (“*x*” value = YAT2150 IC_50_ and “*y*” value = artemisinin IC_50_). Fractional inhibitory concentration (FIC) values were calculated by dividing the IC_50_ of one of the compounds in the mixture by the IC_50_ of the same compound in the 1:0 or 0:1 ratio mixtures.

For stage of growth inhibition analysis, *P. falciparum* cultures were synchronized at ring or trophozoite stages by repeated treatment with 5% sorbitol or 70% Percoll (GE Healthcare, Chicago, IL, US) [79, 81], respectively. Half of each culture remained untreated and the other half was treated with the IC_80_ [27] of YAT2150. At different time points, culture samples were stained with Giemsa and the number of ring, early and mature trophozoites and schizonts was counted by microscopic examination of at least 100 pRBCs for each sample. Pictures were taken with a Nikon Eclipse 50i microscope equipped with a DS-Fi1 camera (Nikon Corporation, Tokyo, Japan).

### Quantitative analysis of protein aggregation in live *P. falciparum* cultures

*P. falciparum* cultures enriched in early stages were treated with the IC_10_ (27 nM) or IC_50_ (90 nM) of YAT2150 or left untreated. After 90 min, 4 h and 30 h, a Percoll purification was done in order to isolate parasitized cells from uninfected RBCs. After Percoll purification, the pellets of late-stage parasites and a control non-infected RBC suspension containing the same proportion of cells as the purified cultures were resuspended in 50 µl of lysis buffer (4.5 mg/ml NaCl in water supplemented with EDTA-free protease inhibitor cocktail, PIC, Hoffman-La Roche, Basel, Switzerland; 1 PIC tablet/10 ml water) and incubated overnight, at 4 °C under stirring, with the objective of releasing their inner content. After this time, lysed samples were spun down and the protein content in the supernatant was quantified with the bicinchoninic acid assay (Thermo Fisher Scientific), following the manufacturer’s instructions. Thirty micrograms of protein from each supernatant was further diluted with PBS to a final volume of 70 µl and plated on a 96-well black plate in triplicates. ThT fluorescence was measured as described above.

### Flow cytometry for cell targeting studies

A non-synchronized *P. falciparum* 3D7 culture was stained with 1 μM YAT2150 and 2 μg/ml of the DNA dye Hoechst 33342. Five microliters of this culture were mixed with 500 μl of PBS and analyzed in a LSRFortessa flow cytometer set up with the five-laser, 20-parameter standard configuration. Forward and side scatter were used in a logarithmic scale to gate the RBC population. Acquisition was configured to stop after recording 30,000 events. Hoechst 33342 and YAT2150 fluorescence levels were detected, respectively, by excitation with 350 and 561 nm lasers, and emissions were collected with 450/50BP and 600LP-610/20BP nm bandpass filters. The fraction of pRBCs containing fluorescein-labeled peptides was also assessed by flow cytometry, in this case exciting with a 488 nm/60 mW laser and collecting the emission with a 525/50BP nm bandpass filter. To avoid fixation artifacts, all the flow cytometry data presented in this work were obtained with live cells.

### Peptide loading into ghost RBCs

Ghost RBCs loaded with various peptides were generated as previously described [53]. Briefly (Fig. 1B), regular RBCs were washed twice using three times their volume of ice-cold 1 × PBS by centrifugation at 200 × *g* for 10 min at 4 °C. After the second washing, the supernatant was removed and the RBC pellet taken up in one volume of ice-cold lysis buffer, 1 mM ATP, 5 mM K_2_HPO_4_ in double deionized water (ddH_2_O; MilliQ system, Millipore Corporation, Burlington, MA, US), containing 10 µM of the peptide to encapsulate. RBCs were incubated with the lysis buffer at 4 °C with gentle stirring for 1 h, when the generated ghost RBCs were spun down and half of the total volume of the sample was substituted by resealing buffer. The final concentration of the buffer after mixing with the sample was 150 mM NaCl, 5 mM MgCl_2_, 1 mM ATP, and 1 mM glutathione. Ghost RBCs were incubated with the resealing buffer for 1 h at 37 °C with gentle stirring. Finally, the samples were washed four times with three times their volume of Roswell Park Memorial Institute 1640 medium (RPMI, Gibco®, Thermo Fisher Scientific) containing L-glutamine and sodium bicarbonate, and supplemented with 5.95 g/ml 2-[4-(2-hydroxyethyl)piperazin-1-yl]ethanesulfonic acid (HEPES). The ghost RBC pellet was taken up in an equal volume of RPMIc: RPMI containing 5 mg/ml Albumax II (Invitrogen, Waltham, MA, US) and 2 mM L-glutamine, and finally stored at 4 °C until further use.

Infection of ghost RBCs or RBCs with *P. falciparum* was performed by establishing a new culture using late-stage parasites purified in 70% Percoll as described elsewhere [79, 81]. Parasites were added to peptide-loaded ghost RBC cultures (Fig. 1B) or regular RBC cultures containing the same proportion of peptide. After 72 h of incubation as described above, the viability of *Plasmodium* cells was assessed by staining parasites in the culture with 2 µg/ml Hoechst 33342 and analyzing the parasitemia by flow cytometry as described above. The % of growth inhibition was calculated comparing the parasitemia in the treated sample with the parasitemia of an untreated control culture, according to the formula: 100 − % survival, where % survival was calculated as follows:

$$\frac{\mathrm{sample}\;\%\;\mathrm{parasitemia}\;-\;\mathrm{initial}\;\%\;\mathrm{parasitemia}}{\mathrm{final}\;\%\;\mathrm{parasitemia}\;\mathrm{of}\;\mathrm{untreated\;control}\;-\;\mathrm{initial}\;\%\;\mathrm{parasitemia}} \times 100$$

where parasitemia was calculated as:

$$\frac{\mathrm{number}\;\mathrm{of}\;\mathrm{pRBCs}}{\mathrm{total}\;\mathrm{number}\;\mathrm{of}\;\mathrm{parasitized}\;+\;\mathrm{na\ddot{i}ve}\;\mathrm{RBCs}} \times 100$$

### Fluorescence microscopy

For YAT2150 staining, a *P. falciparum* 3D7 culture was incubated in RPMIc for 30 min at 37 °C with 4.5 µM of the compound and 4 μg/ml of Hoechst 33342. For colocalization studies, 0.5 µM of ER Tracker™ Green (BODIPY™ FL Glibenclamide, Thermo Fisher Scientific) was included in the solution. Cells were placed in an 8-well LabTek™ II chamber slide system (Thermo Fisher Scientific), rinsed with warm PBS and diluted 1:20 for their observation in a Leica TCS SP5 confocal microscope (Leica Camera, Mannheim, Germany) equipped with a × 63 objective of 1.4 NA. Hoechst 33342 was excited with a diode laser at 405 nm, ER Tracker Green with the 488 nm line of an argon laser, and YAT2150 with a diode-pumped solid-state laser at 561 nm. The corresponding fluorescence emissions were collected in the ranges of, respectively, 415–460, 490–590, and 600–700 nm. The subcellular localization in ghost pRBCs of fluorescein-labeled aggregative peptides was done in cultures that had been grown in ghost RBCs loaded with 10 µM peptides labeled in their N-ter ends with 5/6-FAM. Colocalization was evaluated as above but in this case using 0.5 µM of either ER Tracker™ Red (BODIPY™ TR Glibenclamide, Thermo Fisher Scientific) or LysoTracker™ Red DND-99 (Thermo Fisher Scientific) in addition to Hoechst 33342. Emissions of ER Tracker Red and LysoTracker Red (both excited with a diode-pumped solid-state laser at 561 nm) were collected between 590 and 680 nm whereas the peptide signal (following excitation at 488 nm) was detected in the 490 to 550 nm range. To avoid crosstalk between the different fluorescence signals, sequential line scanning was performed. To quantify Manders’ overlap coefficient [82], images were analyzed using the Just Another Colocalization Plugin (JACoP, [83]) in the Fiji software [84]. To avoid fixation artifacts, all the fluorescence microscopy data presented in this work were obtained with live cells.

### LC–MS/MS analysis of aggregative proteins from ring stage parasites

For the isolation of aggregative proteins from ring stage parasites, 80 ml of a *P. falciparum* preparation containing approximately 4 × 10^9^ early-stage parasites that had been sorbitol-synchronized from in vitro cultures were washed with sterile PBS and spun down (300 × *g*, 5 min), storing the resulting cell pellet at − 80 °C until performing LC–MS/MS analysis as previously described [3].

### Dot blots and Western blots

Cultures of the *P. falciparum* 3D7 strain were sorbitol-synchronized in ring stages, and after 24 h were treated for 90 min with YAT2150 concentrations ranging from 33 nM to 33 µM, or for 24 h with 90 nM YAT2150. After that time, cultures were spun down and pellets were washed once with ice-cold PBS supplemented with EDTA-free PIC (1 PIC tablet/10 ml PBS). For anti-ubiquitin Western blots, PBS was also supplemented with 20 mM *N*-ethylmaleimide. Washed parasite pellets were treated with 0.15% saponin at 4 °C for 15 min and washed again by centrifugation (10,000 × *g*, 15 min, 4 °C) with appropriately supplemented PBS until no hemoglobin was observed in the supernatant. Protein extracts were quantified with the bicinchoninic acid assay. For dot blots, 4-μl drops of saponin extract containing 0.5 or 1 mg/ml protein were spotted on a nitrocellulose membrane. Once protein extracts were completely absorbed by the membranes, these were incubated for 3 h in blocking solution: 5% milk powder in tris-buffered saline (0.15 M NaCl, 20 mM tris-base, pH 7.6) supplemented with 0.1% Tween-20 (TBS-Tween). The blocked membranes were washed 3 × 5 min with TBS-Tween and incubated overnight at 4 °C with rabbit polyclonal anti-amyloid fibrils OC antibody (AB2286, Millipore Corporation) diluted 1:500 in blocking solution or with mouse monoclonal anti-spectrin α/β (S3396, Sigma-Aldrich Corporation) diluted 1:10,000 in TBS-Tween. For Western blots, 15 μg of saponin-extracted proteins was incubated for 5 min at 95 °C diluted in Laemmli solution (0.14 M SDS, 0.125 M tris–HCl, pH 6.8, 20% glycerol, 10% 2-mercaptoethanol, 3 mM bromophenol blue) and resolved by SDS–polyacrylamide gel electrophoresis in 12% bis–tris acrylamide (Bio-Rad Laboratories, Inc., Hercules, CA, US) gels run at 80 V until samples entered the resolving gel and at 120 V afterwards. Proteins were transferred from the gel to polyvinylidene difluoride membranes activated with methanol. After transference, membranes were blocked with blocking solution for 1 h at RT, washed 3 × 5 min with TBS-Tween and probed overnight at 4 °C with rabbit polyclonal anti-ubiquitin antibody (#3933, Cell Signaling Technology, Inc., Danvers, MA, US) diluted 1:1000 in blocking solution, or with mouse monoclonal anti-spectrin α/β diluted 1:10,000 in TBS-Tween. Then, membranes were washed 5 times with TBS-Tween and incubated for both dot blot and Western blot during 1 h with either goat anti-rabbit (#12–348, Upstate Biotechnology, Inc., Lake Placid, NY, US) or goat anti-mouse (#145660, Amersham Life Science, Inc., Amersham, UK) IgG-horseradish peroxidase conjugate diluted 1:10,000 in TBS-Tween. After 4 washes with TBS-Tween and one last wash with TBS, peroxidase substrate (ECL Prime Western Blotting Detection Reagent, Amersham Life Science, Inc.) was poured on the membrane and chemiluminescent signal was measured in a LAS 4000 reader (ImageQuant TL, GE Healthcare, Chicago, IL, US) at different exposure times.

### Transmission electron microscopy (TEM)

A carbon-coated copper grid was deposited for 30 min on top of a 50-µl drop of 25 µM peptide solutions prepared as explained above. Then, the excess liquid was removed with filter paper and the grid was placed on top of a ddH_2_O drop for 30 s and finally negatively stained for 2 min with 20 µl of 2% uranyl acetate. Samples were observed using a JEM 1010 transmission electron microscope (JEOL Ltd., Tokyo, Japan). Images were acquired using a CCD Orius camera (Gatan, Inc., Pleasanton, CA, US).

### Correlative light and electron microscopy (CLEM)

A 0.5% parasitemia RBC culture was prepared for CLEM by allowing its binding to concanavalin as described [85]. Briefly, a µ-Dish 35 mm, High, Grid*-*500 (ibidi GmbH, Gräfelfing, Germany) was coated for 20 min at 37 °C with a 50 mg/ml concanavalin A solution in ddH_2_O and wells were rinsed with pre-warmed PBS before parasite seeding. *P. falciparum*-infected RBCs washed twice with PBS were deposited into the dish and incubated for 10 min at 37 °C; afterwards, unbound RBCs were washed away with three PBS rinses. Seeded RBCs were then incubated with 3 µM YAT2150, and nuclei were counterstained with 2 µg/ml Hoechst 33342. The preparation was observed with a Zeiss LSM880 confocal microscope (Carl Zeiss, Jena, Germany), with respective λex/em for YAT2150 and Hoechst 33342 of 405/415–520 and 561/565–600 nm. Images were obtained from areas corresponding to a specific coordinate of the dish-grid by tile scans that were stitched into larger mosaics. A bright-field image facilitated the recognition of the grid coordinates from the plate where the cells selected for CLEM were located. After confocal image acquisition, cells were washed three times with TEM fixation buffer (2% paraformaldehyde and 2.5% glutaraldehyde in PBS) for 5 min each. Then, the fixation buffer was changed to 1% osmium tetroxide and 0.8% potassium ferricyanide in fixation buffer and incubated at 4 °C for 45 min, followed by three 5-min washes with ddH_2_O. Then, a dehydration procedure was performed by gradually increasing ethanol concentration: 50% (10 min), 70% (10 min), 80% (10 min), 90% (5 min, 3 ×), 96% (5 min, 3 ×), and 100% (5 min, 3 ×). At this point, the plastic part of the dish was carefully separated from the crystal part containing the samples, which was embedded in Spurr resin by successive incubations with different proportions of resin/ethanol, starting with 1/3 for 1 h, 1/1 for 1 h, 3/1 for 1 h, and 1/0 overnight. After the embedding procedure, a BEEM® capsule containing polymerized Spurr resin was filled with a small volume of liquid resin in order to obtain an interphase in which the dish was placed. The BEEM® capsule was incubated at 70 °C for 72 h, and the crystal part of the dish was removed by alternatively immersing samples in liquid nitrogen and boiling water. When the crystal was broken, cells remained attached to the resin, which was further cut in a microtome with a diamond blazer in order to obtain 100-nm-thick resin slides, which were mounted on a carbon-coated copper grid and negatively stained with 2% uranyl acetate for 2 min and washed with ddH_2_O for 1 min. Samples were observed in a JEM 1010 transmission electron microscope. Images were processed for CLEM analysis using the CORRELIA plugin [86] in the Fiji software (version 2.0.0-pre-8) [84].

### Hemozoin formation assay

In vitro hemozoin formation assays were performed as explained elsewhere [87, 88] with minor modifications. A stock of 4.5 mg/ml hemin chloride in DMSO was further diluted to obtain a solution of 0.036 mg/ml in 0.1 M acetate buffer (pH 4.8) containing 0.015 mg/ml of Tween-20. This solution was distributed in Eppendorf tubes, and chloroquine or YAT2150 were added at different concentrations. An untreated hemin sample and controls containing drugs but not hemin were also prepared. To monitor the initial turbidimetry and free hemin, absorbance was measured, respectively, at 630 and 415 nm (Infinite Nano + multimode microplate reader) in triplicates, and tubes were vortexed and incubated protected from light in a ThermoMixer® (37 °C, 2 h, 700 rpm). After 2 h, samples were left at RT for 1 h in the dark and then centrifuged (10 min, 21,300 × *g*) to precipitate hemozoin crystals. The supernatant of each sample was recovered and plated in triplicates (150 µl/well, 96-well plates) and absorbance was read again. The amount of free hemin in each sample was calculated (A_415_–A_630_) and subtracted from the free drug control.

### Gametocyte assays

Cultures of the *P. falciparum NF54-gexp02-Tom* strain (developed and authenticated by Portugaliza et al. [89] and kindly provided by Prof. Alfred Cortés) were maintained in standard conditions in RPMI medium supplemented with 0.5% Albumax II and 2 mM choline, synchronized in ring stages with sorbitol lysis, and diluted to 2% parasitemia. To trigger sexual conversion, choline was removed from the medium and cultures were maintained in the same conditions for 48 h after synchronization (cycle 0). In the next cycle (cycle 1), parasites were treated with 50 mM *N*-acetylglucosamine (GlcNac) in order to kill asexual stages, and maintained in RPMI supplemented with 10% human serum. Medium was refreshed daily and GlcNac was kept during 4 days. To determine the effect of YAT2150 and DONE3TCl in early gametocytes, the culture was distributed in triplicates (200 µl/well, 96-well plates) and drugs were added in cycle 1 and maintained for 48 h in the culture. Controls of untreated parasites as well as of parasites treated with a lethal dose of chloroquine were prepared. Gametocytemia was monitored daily by light microscopy until the majority of parasites (~ 90%) could be identified as stage V gametocytes. At that point, Giemsa smears of each well were prepared and mature gametocytes were manually counted (10,000 cells were counted for each replica by two investigators blinded to group assignment). To test the effect of YAT2150 and DONE3TCl on mature gametocytes, cultures were grown for 14 days, when the majority of the parasites could be identified as stage V gametocytes. Afterwards, the culture was treated for 48 h with the drugs and the gametocytemia determined as above.

### In vitro activity against *P. berghei* hepatic stages

The in vitro activity of YAT2150 and DONE3TCl against the liver stages of *P. berghei* (obtained from Leiden University Medical Centre, Leiden, The Netherlands) infection was assessed as previously described [90]. Briefly, Huh7 cells (Cenix BioScience GmbH, Dresden, Germany) were routinely cultured in RPMI supplemented with 10% (v/v) fetal bovine serum (FBS), 1% (v/v) glutamine, 1% (v/v) penicillin/streptomycin, 1% non-essential amino acids, and 10 mM HEPES. For drug screening experiments, Huh7 cells were seeded at 1 × 10^4^ cells/well of a 96-well plate and incubated overnight at 37 °C with 5% CO_2_. Stock solutions of test compounds (10 mM) were prepared in DMSO and serially diluted in infection medium, i.e., culture medium supplemented with gentamicin (50 μg/ml) and amphotericin B (0.8 μg/ml), in order to obtain the test concentrations. On the day of the infection, the culture medium was replaced by serial dilutions of test compounds and incubated for 1 h at 37 °C with 5% CO_2_. Next, 1 × 10^4^ firefly luciferase-expressing *P. berghei* sporozoites, freshly isolated from the salivary glands of female infected *Anopheles stephensi* mosquitoes (reared from eggs originally obtained from the Radboud University Medical Centre, Nijmegen, The Netherlands), were added to the cultures, and plates were centrifuged at 1800 × *g* for 5 min at room temperature and incubated at 37 °C with 5% CO_2_. To assess the effect of each compound concentration on cell viability, cultures were incubated with Alamar Blue (Invitrogen, Waltham, MA, US) at 46 h post infection, according to the manufacturer’s recommendations. The parasite load was then assessed by a bioluminescence assay (Biotium, Fremont, CA, US), using a multi-plate reader, Infinite M200 (Tecan Trading AG). Nonlinear regression analysis was employed to fit the normalized results of the dose–response curves, and IC_50_ values were determined using GraphPad Prism 6.0 (GraphPad Software, La Jolla, CA, US).

### In vitro toxicity assays

Human umbilical vein endothelial cells (HUVEC; CRL-1730 American Type Culture Collection, Manassas, VA, US) were plated at 5000 cells/well in 96-well plates and grown in Medium 199 with Earle’s salts supplemented with 10% FBS and 1% penicillin/streptomycin for 24 h at 37 °C in 5% CO_2_. After that, the medium was substituted by 100 µl of drug-containing culture medium without FBS, and incubation was resumed for 48 h. Ten microliters of 4-[3-(4-iodophenyl)-2-(4-nitrophenyl)-2*H*-5-tetrazolio]-1,3-benzene disulfonate labeling reagent (WST-1) was added to each well, and the plate was incubated in the same conditions for 2 h. After thoroughly mixing by pipetting up and down, the absorbance of the samples was measured at 440 nm using a Benchmark Plus microplate reader (Bio Tek, Agilent Technologies, Santa Clara, CA, US). WST-1 in the absence of cells was used as blank, and samples were prepared in triplicate for each experiment. Percentages of viability were obtained using non-treated cells as control of survival. The compound’s concentration required for the reduction of cell viability by 50% was defined as CC_50_. The in vitro selectivity index was defined as CC_50_/IC_50_.

### In vivo toxicity assays

In vivo assays were done at the animal facility of the *Parc Científic de Barcelona* (PCB). BALB/c female and male mice (BALB/cAnNRj, 7 weeks old, Janvier Laboratories, Le Genest-Saint-Isle, France) were maintained with unlimited access to food and water under standard environmental conditions (20–24 °C and 12/12 h light/dark cycle). Three 100-µl doses of a drug solution prepared to administer 0.0959, 0.3069, and 0.9822 mg YAT2150/kg were tested in a total number of 6 mice/drug dose. First, the lowest dose was intravenously injected to one female and one male mouse. An oxygen stream of 4% isoflurane was used to anesthetize the mice, which were then maintained during the whole injection procedure (less than 3 min) with 2.5% isoflurane. After the administration, mice were observed and different parameters related to their behavior (lethargy, motility alterations, seizures, coma, automutilation, aggressiveness, vocalizations, stereotyped movements) and physical conditions (pain, respiratory disturbances, tachycardia or bradycardia, dehydration, hair loss, body weight loss, dermatitis, bad hygiene, pruritus, tearing) were followed. If after 48 h no deleterious effects were observed, the following dose was administered to two other male and female mice. All mice were observed for 14 days after treatment in order to detect long-term side effects.

### In silico analysis

Protein abundance and aggregation propensity were calculated and plotted as elsewhere described [91]. Briefly, abundance (C) was calculated as the log_10_ of the protein concentration values obtained from PaxDb [92], which were normalized by rescaling them between 0 and 1:

1 $$C=\frac{\left(Ci-\mathrm{min}(Ci\dots Cn)\right)}{\left(\mathrm{max}\left(Ci\dots Cn\right)-\mathrm{min}(Ci\dots Cn)\right)},$$

where (*Ci…Cn*) is each value of protein concentration from the dataset, *Cmin* is the minimum value of protein concentration from the dataset, and *Cmax* is the maximum value of protein concentration from the dataset.

Aggregation tendency (A) was obtained using the TANGO algorithm, which estimates the cross-beta aggregation propensity in peptides and denatured proteins [93]. For the estimation, TANGO parameters were set at pH 7.4, 37 °C and 0.25 mM ionic strength, using the output parameter “AGG,” which was then normalized in the same manner by rescaling the values between 0 and 1:

2 $$A=\frac{\left(Ai-\mathrm{min}\left(Ai\dots An\right)\right)}{\left(\mathrm{max}\left(Ai\dots An\right)-\mathrm{min}\left(Ai\dots An\right)\right)},$$

where (*Ai…An*) is each value of protein aggregation from the dataset according to the “AGG” parameter of TANGO, *Amin* is the minimum value of protein aggregation from the dataset, and *Amax* is the maximum value of protein aggregation from the dataset.

Peptide aggregation scores were obtained with the WALTZ algorithm [54], designed to predict amyloidogenic regions inside proteins. The values expressed correspond to the average score per residue given by the algorithm.

### Statistical analysis

Except where otherwise indicated, all statistical analyses were performed using GraphPad Prism 9 (GraphPad Software). The normal distribution of the obtained data was assessed by various normality tests (Shapiro–Wilk, Anderson–Darling, D’Agostino-K and Chen-Shapiro), and a two-sided test of variance was performed. Afterwards, samples were analyzed by two-sided Student’s *t* test. All tests were accomplished at the 0.05 significance level cut-off.

## Supplementary Information

**Additional file 1:** **Figures S1-S12** and **Tables S1-S9.** **Figure S1.** Analysis of the aggregation of six peptides present in *P. falciparum* proteins. **Figure S2.** Flow cytometry analysis of the colocalization with RBCs and pRBCs of fluorescein-labeled LMWP-conjugated peptides. **Figure S3.** Flow cytometry analysis of the colocalization with RBCs and pRBCs of fluorescein-labeled TAT-conjugated peptides. **Figure S4.** Flow cytometry analysis of the colocalization with RBCs and pRBCs of fluorescein-labeled TP2-conjugated peptides. **Figure S5.** Flow cytometry analysis of the parasitemia, 72 h post-infection, in *P. falciparum* cultures grown in regular and ghost RBCs. **Figure S6.** Aggregative proteins found in late and early *P. falciparum* blood stages represented according to their abundance and aggregation propensity normalized relative to the whole proteome. **Figure S7.** In vitro analysis of the aggregation of KDLLF, KVVNI and derived peptides. **Figure S8.** Confocal fluorescence microscopy analysis of the presence of the fluorescein-labeled peptides KDLLF and KVVNI, and of their LMWP elongations, in ghost pRBCs. **Figure S9.** Effect of YAT2150 on pre-aggregated Aβ40. **Figure S10.** ThT analysis of the effect of YAT2150 on the in vitro aggregation of aggregative peptides present in *P. falciparum* proteins. **Figure S11.** Determination of protein aggregation in live *P. falciparum* cultures. **Figure S12.** Hemozoin formation assay. **Table S1.** Aggregative peptides selected from the pool of 369 proteins resisting dissolution in 0.1% SDS identified in Biosca et al., 2020. **Table S2.** Growth inhibition assay in regular *P. falciparum* cultures of fluorescein-labeled aggregative peptides conjugated to CPPs. **Table S3.** Growth inhibition assay in ghost RBC-enriched *P. falciparum* cultures treated with 10 µM non-modified aggregative peptides. **Table S4.** Cytotoxicity assay in HUVEC cultures of the aggregative peptides which at 10 µM reduced by > 20% *P. falciparum* growth in ghost pRBC cultures. **Table S5.** Early stage *P. falciparum* proteins resisting dissolution in 0.1% SDS. **Table S6.** Aggregative peptides identified in *P. falciparum* E3 ubiquitin-protein ligase (C0H4K6). **Table S7.*** P. falciparum* proteins in which the peptides KDLLF and KVVNI are present. **Table S8.** Growth inhibition assay in ghost RBC-enriched *P. falciparum* cultures treated with 10 µM KDLLF and KVVNI peptides. **Table S9.** In vitro toxicity in HUVEC cultures of amyloid pan-inhibitors.

**Additional file 2.** Individual data values.

**Additional file 3.** Original, uncropped blots from Fig. 9.

## Data Availability

All data generated or analyzed during this study are included in this published article and its supplementary information files.
