# Supplementary material for: The protein aggregation inhibitor YAT2150 has potent antimalarial activity in Plasmodium falciparum in vitro cultures
Source: BMC Biol. 2022 Oct 22;20:197. doi: 10.1186/s12915-022-01374-4 (PMC9587658; doi:10.1186/s12915-022-01374-4)
Supplement: Supplementary file 1 — Additional file 1: Figures S1-S12 and Tables S1-S9. Figure S1. Analysis of the aggregation of six peptides present in P. falciparum proteins. Figure S2. Flow cytometry analysis of the colocalization with RBCs and pRBCs of fluorescein-labeled LMWP-conjugated peptides. Figure S3. Flow cytometry analysis of the colocalization with RBCs and pRBCs of fluorescein-labeled TAT-conjugated peptides. Figure S4. Flow cytometry analysis of the colocalization with RBCs and pRBCs of fluorescein-labeled TP2-conjugated peptides. Figure S5. Flow cytometry analysis of the parasitemia, 72 h post-infection, in P. falciparum cultures grown in regular and ghost RBCs. Figure S6. Aggregative proteins found in late and early P. falciparum blood stages represented according to their abundance and aggregation propensity normalized relative to the whole proteome. Figure S7. In vitro analysis of the aggregation of KDLLF, KVVNI and derived peptides. Figure S8. Confocal fluorescence microscopy analysis of the presence of the fluorescein-labeled peptides KDLLF and KVVNI, and of their LMWP elongations, in ghost pRBCs. Figure S9. Effect of YAT2150 on pre-aggregated Aβ40. Figure S10. ThT analysis of the effect of YAT2150 on the in vitro aggregation of aggregative peptides present in P. falciparum proteins. Figure S11. Determination of protein aggregation in live P. falciparum cultures. Figure S12. Hemozoin formation assay. Table S1. Aggregative peptides selected from the pool of 369 proteins resisting dissolution in 0.1% SDS identified in Biosca et al., 2020. Table S2. Growth inhibition assay in regular P. falciparum cultures of fluorescein-labeled aggregative peptides conjugated to CPPs. Table S3. Growth inhibition assay in ghost RBC-enriched P. falciparum cultures treated with 10 µM non-modified aggregative peptides. Table S4. Cytotoxicity assay in HUVEC cultures of the aggregative peptides which at 10 µM reduced by > 20% P. falciparum growth in ghost pRBC cultures. Table S5. Early stage P. falcipar [file 12915_2022_1374_MOESM1_ESM.docx]

Supporting Information for

**The protein aggregation inhibitor YAT2150 has potent antimalarial activity in *Plasmodium* *falciparum in vitro* cultures**

Inés Bouzón-Arnáiz, Yunuen Avalos-Padilla, Arnau Biosca, Omar Caño-Prades, Lucía Román-Álamo, Javier Valle, David Andreu, Diana Moita, Miguel Prudêncio, Elsa M. Arce, Diego Muñoz-Torrero, Xavier Fernàndez-Busquets*

*Corresponding author. Email: xfernandez_busquets@ub.edu


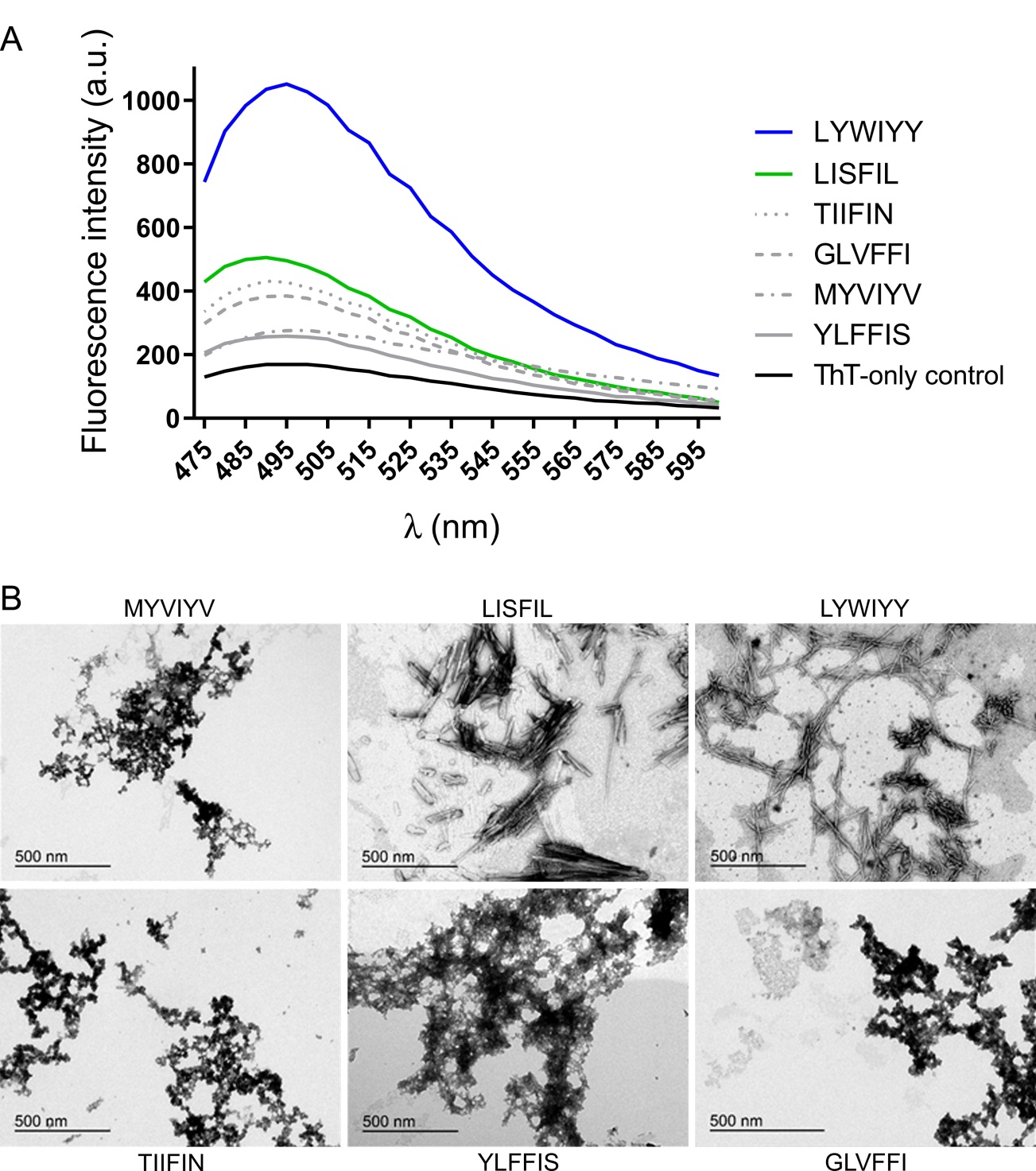


Figure S1. Analysis of the aggregation of six peptides present in *P. falciparum* proteins. (A) Thioflavin T fluorescence analysis. a.u.: arbitrary units. The mean fluorescence intensity value of each sample in each wavelength is represented. (B) Transmission electron microscopy analysis.


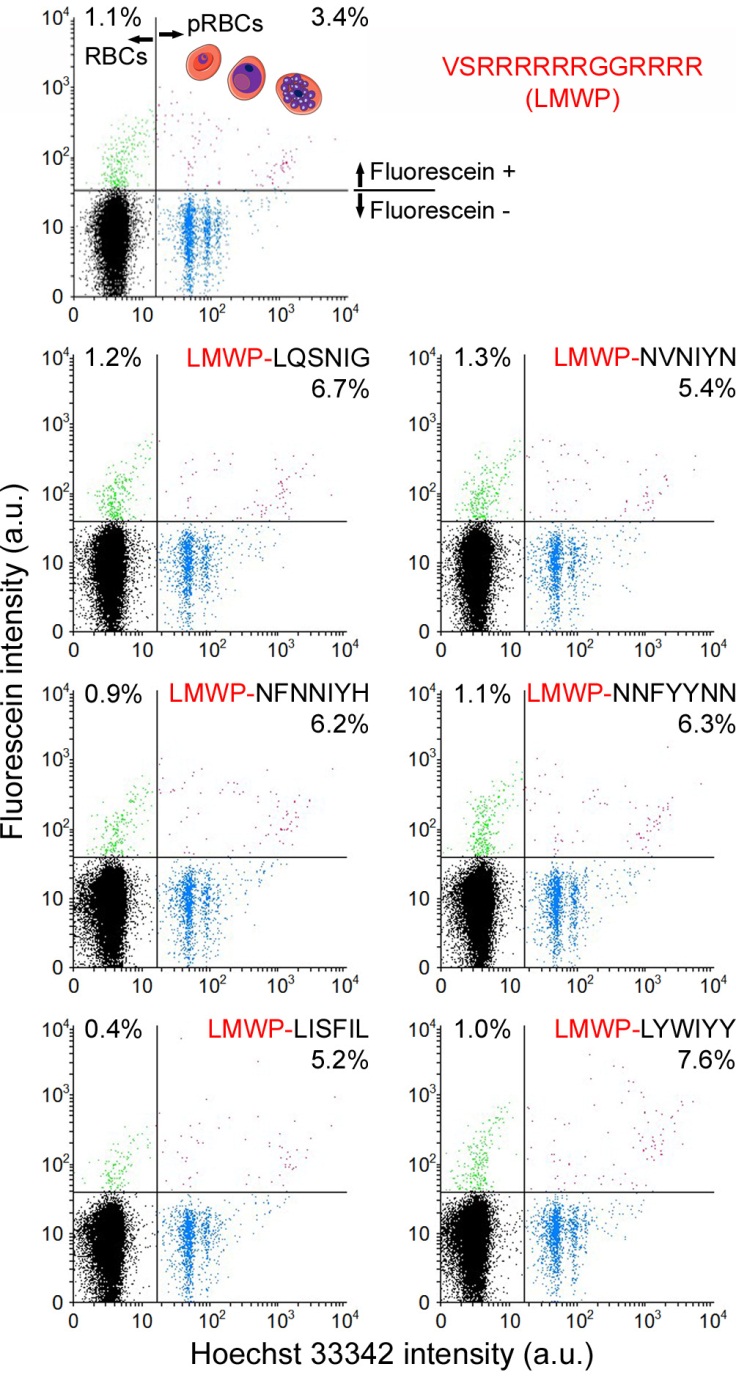


Figure S2. Flow cytometry analysis of the colocalization with RBCs and pRBCs of fluorescein-labeled LMWP-conjugated peptides. Percentages indicate the proportion of RBCs and pRBCs positive for fluorescein. a.u.: arbitrary units.


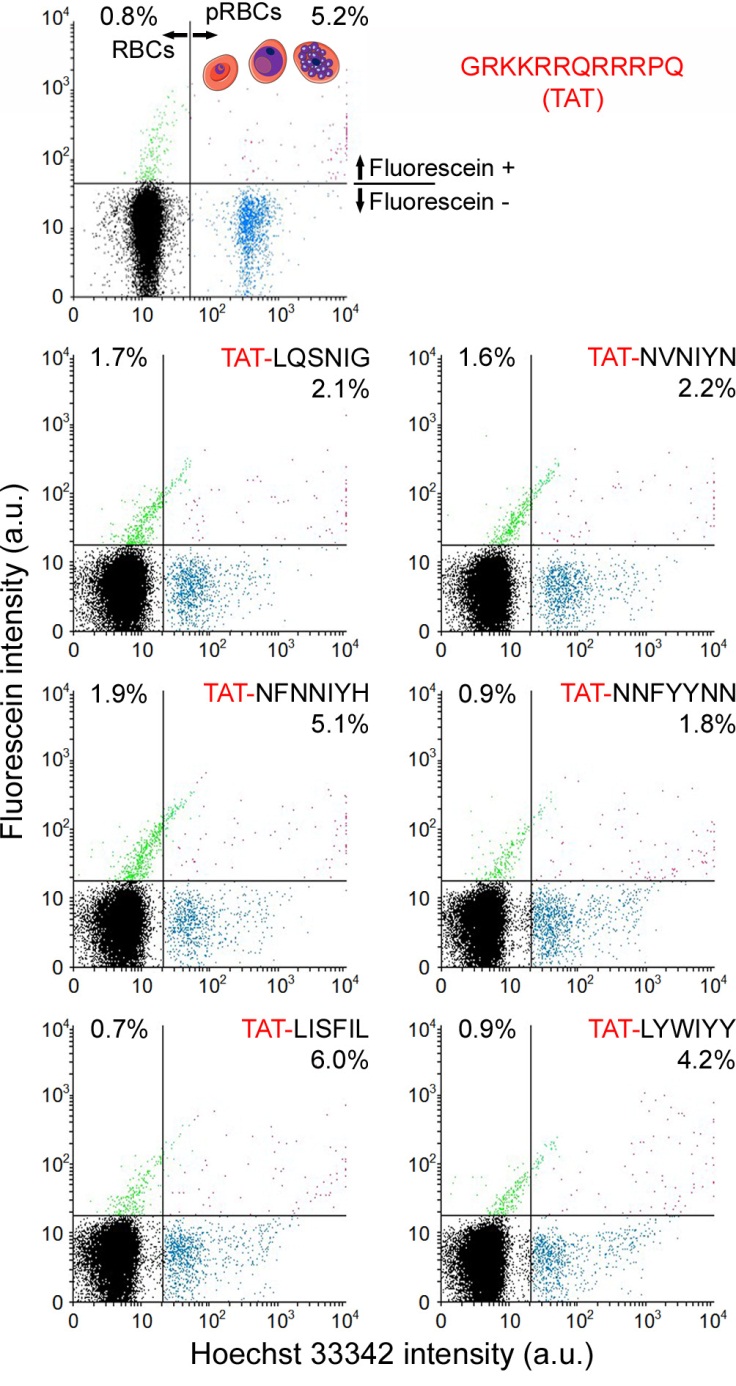


**Figure S3. Flow cytometry analysis of the colocalization with RBCs and pRBCs of fluorescein-labeled TAT-conjugated peptides.** Percentages indicate the proportion of RBCs and pRBCs positive for fluorescein. a.u.: arbitrary units.


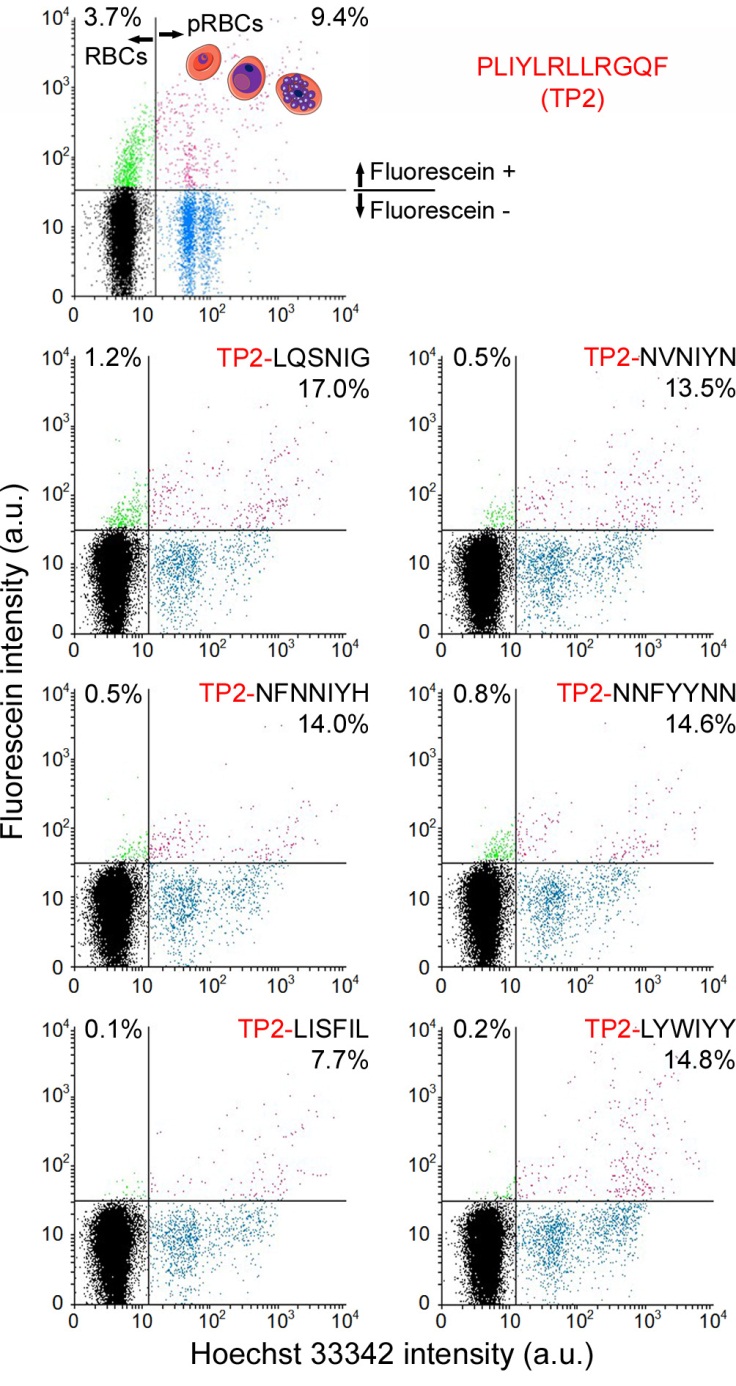


**Figure S4. Flow cytometry analysis of the colocalization with RBCs and pRBCs of fluorescein-labeled TP2-conjugated peptides.** Percentages indicate the proportion of RBCs and pRBCs positive for fluorescein. a.u.: arbitrary units.


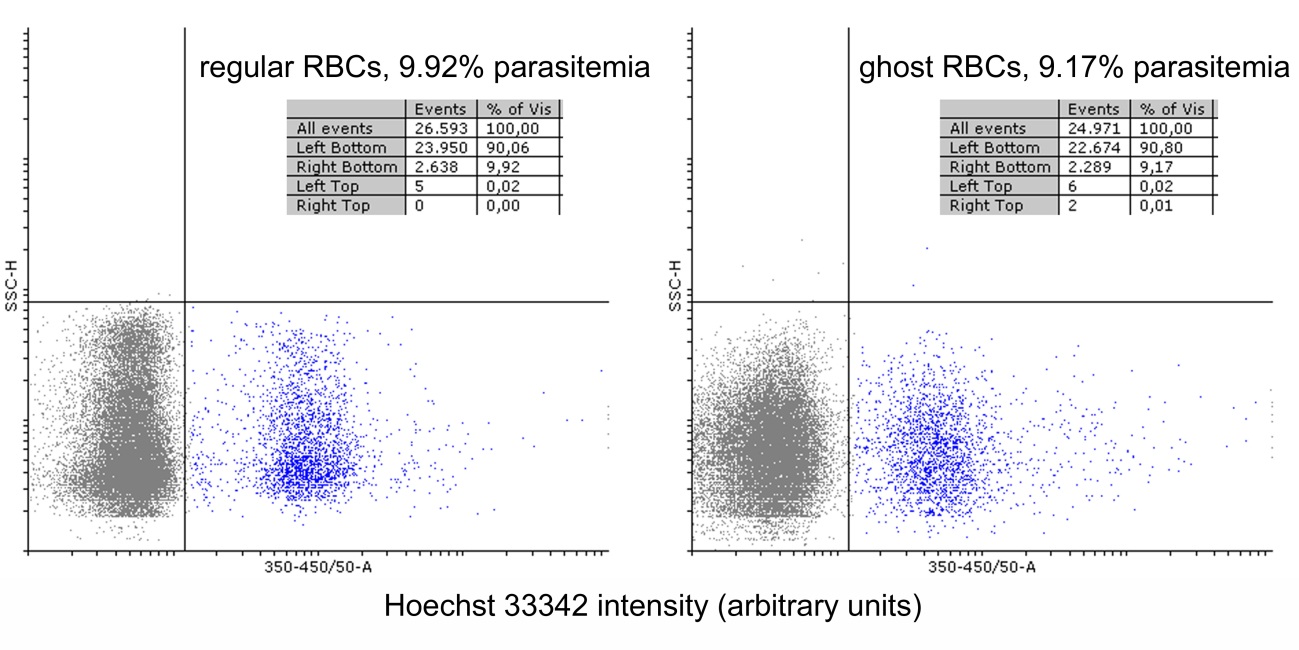


**Figure S5. Flow cytometry analysis of the parasitemia, 72 h post-infection, in *P. falciparum* cultures grown in regular and ghost RBCs.**


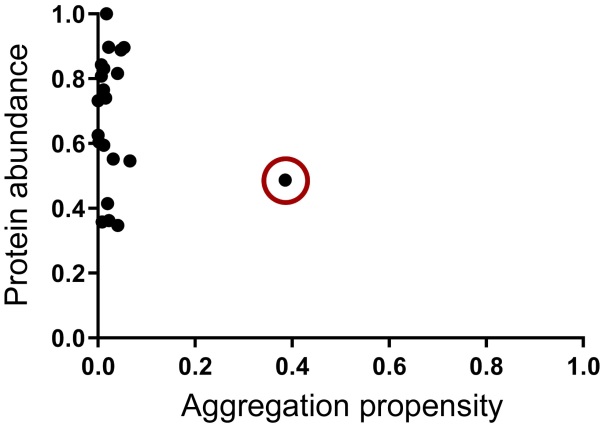


**Figure S6. Aggregative proteins found in late and early *P. falciparum* blood stages represented according to their abundance and aggregation propensity normalized relative to the whole proteome.** Protein aggregation propensity was calculated with the TANGO algorithm. The red circumference indicates E3 ubiquitin-protein ligase (UniProt ID: C0H4K6).


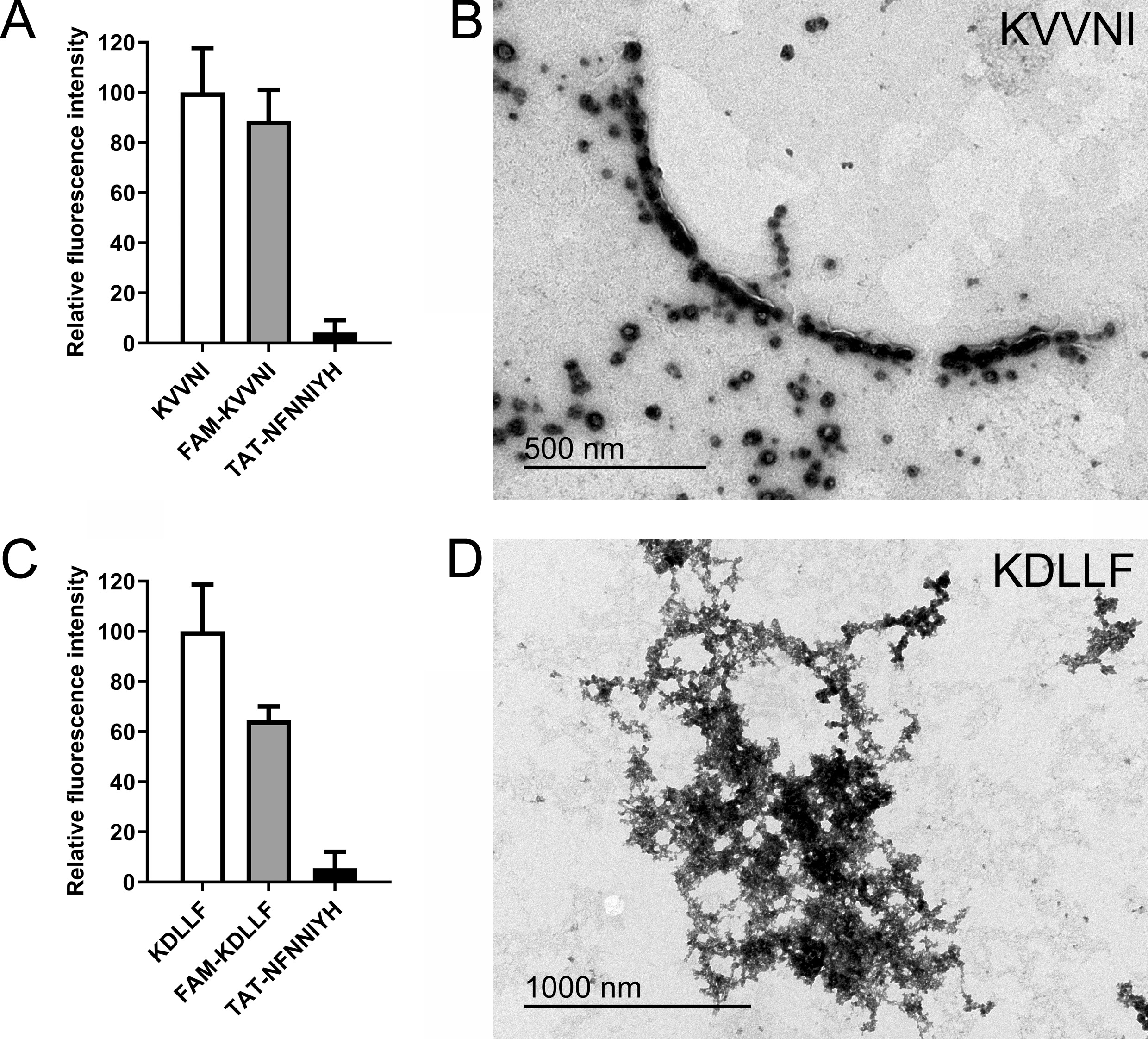


**Figure S7. *In vitro* analysis of the aggregation of KDLLF, KVVNI and derived peptides.** (**A**,**C**) ProteoStat® aggregation assay. TAT-NFNNIYH is included as a negative control of a low-aggregative peptide. Bars represent the mean ± SD. (**B**,**D**) Transmission electron microscopy analysis.

**
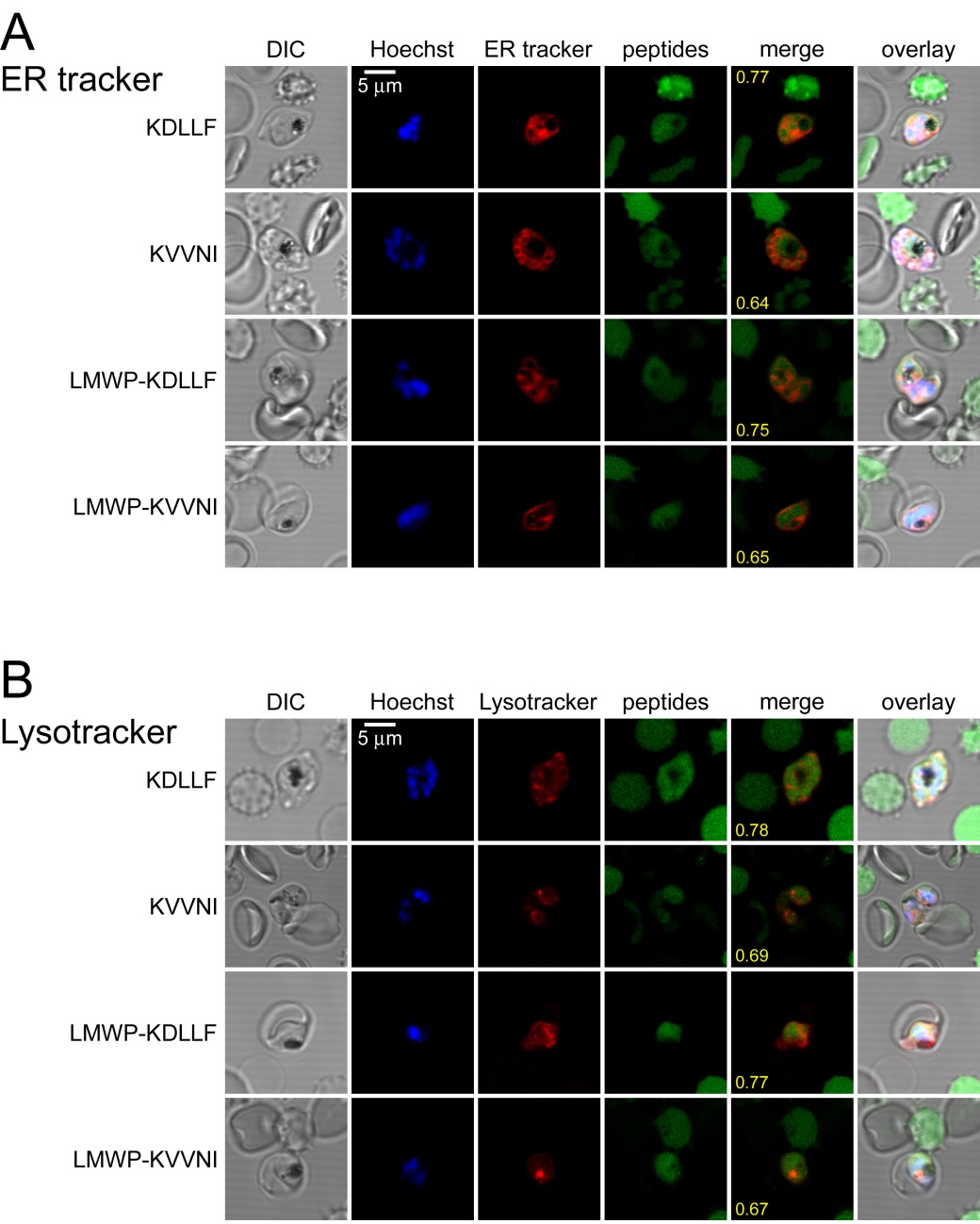
**

**Figure S8. Confocal fluorescence microscopy analysis of the presence of the fluorescein-labeled peptides KDLLF and KVVNI, and of their LMWP elongations, in ghost pRBCs.** (**A**) Colocalization analysis with the cytosolic marker ER Tracker™ Red. (**B**) Colocalization analysis with the digestive vacuole marker LysoTracker™ Red DND-99. The merge images correspond to red and green channels only. Manders’ overlap correlation coefficients are indicated in yellow digits.


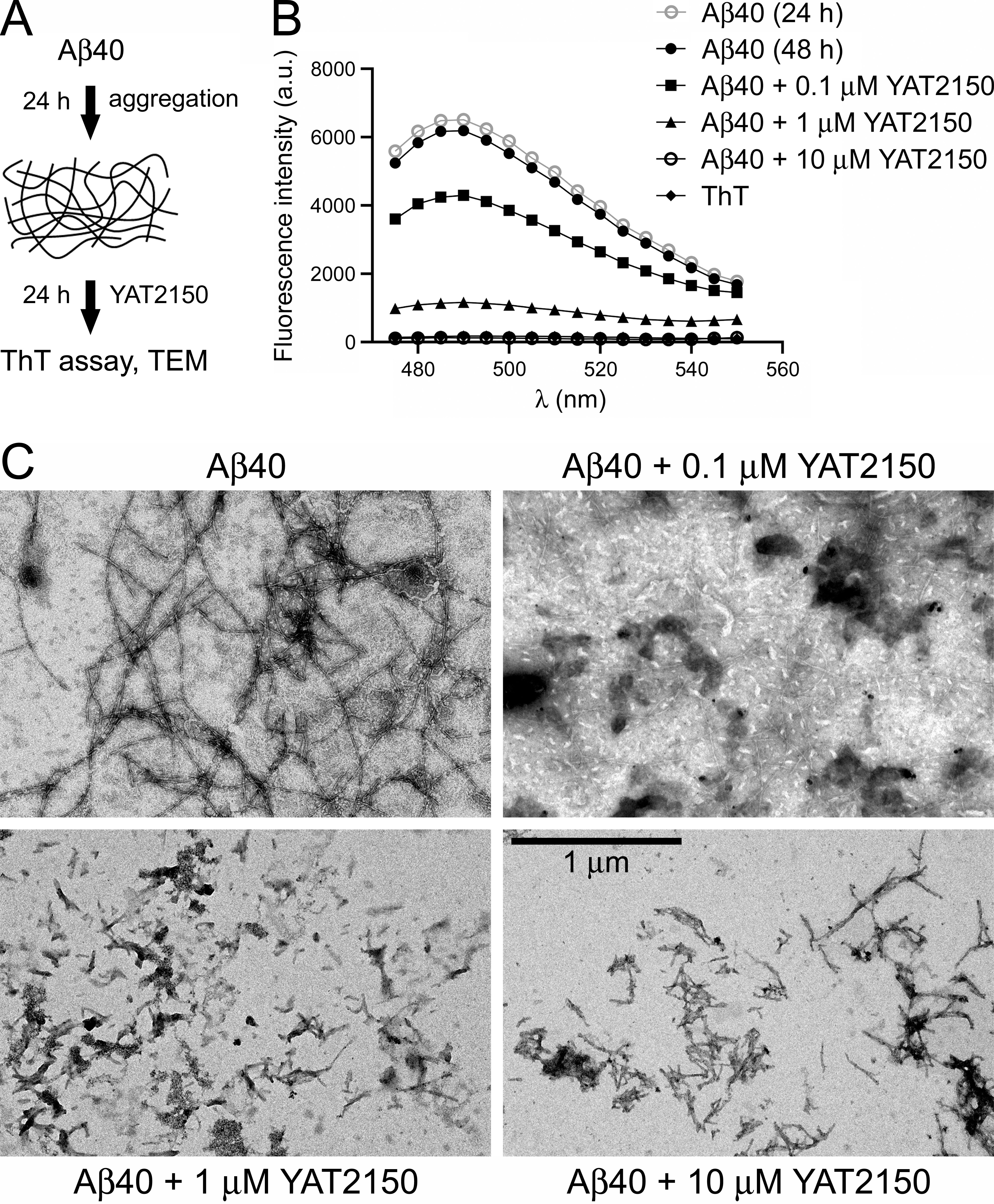


**Figure S9. Effect of YAT2150 on pre-aggregated Aβ40.** (**A**) Scheme of the assay. (**B**) ThT fluorescence assay. a.u.: arbitrary units. The mean fluorescence intensity value of each sample in each wavelength is represented. (**C**) TEM analysis of the samples after 48 h of incubation.


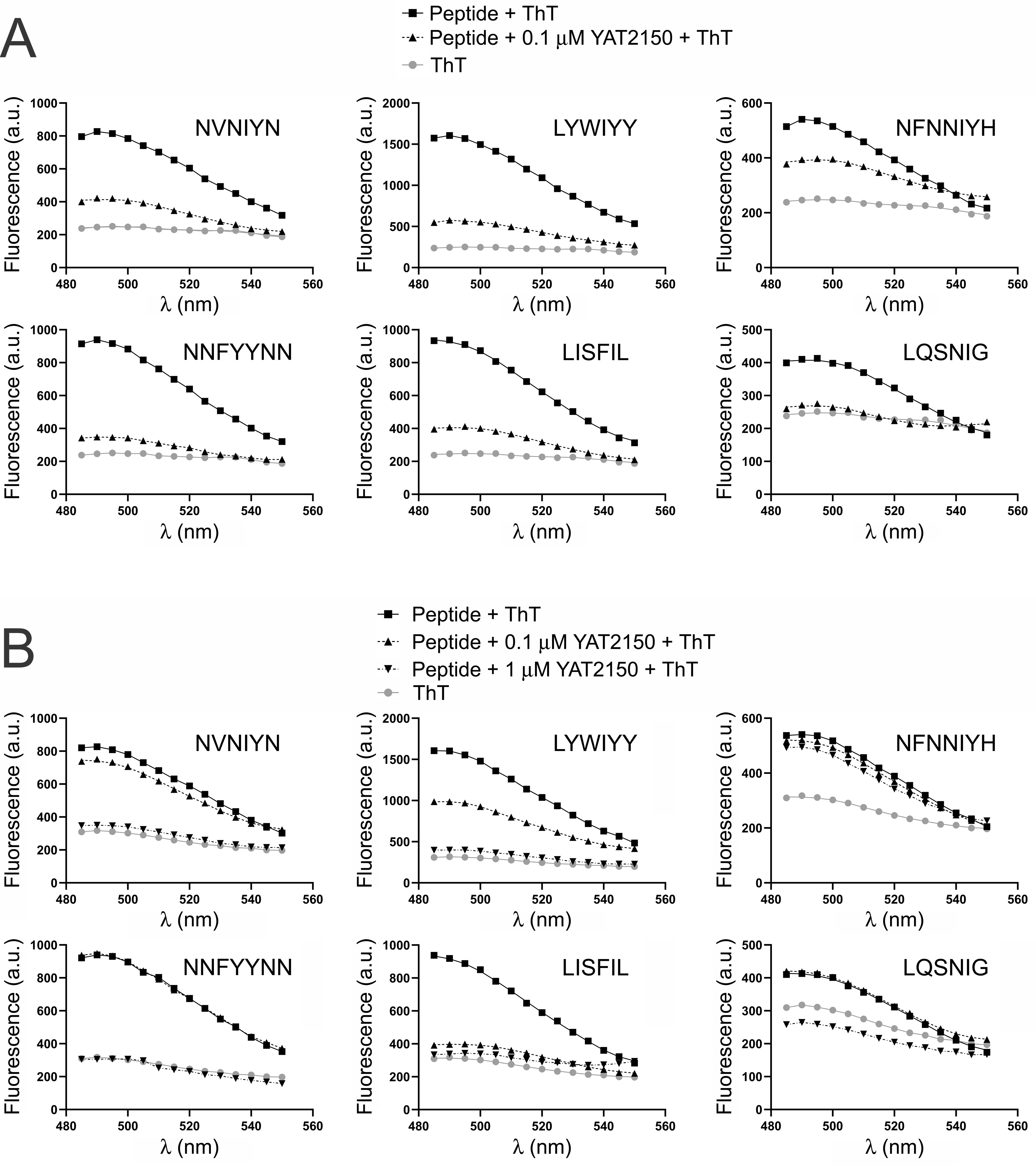


**Figure S10. ThT analysis of the effect of YAT2150 on the *in vitro* aggregation of aggregative peptides present in *P. falciparum* proteins.** (**A**) Inhibition of aggregation assay. (**B**) Disaggregation assay. In both panels the mean fluorescence intensity value of each sample in each wavelength is represented.


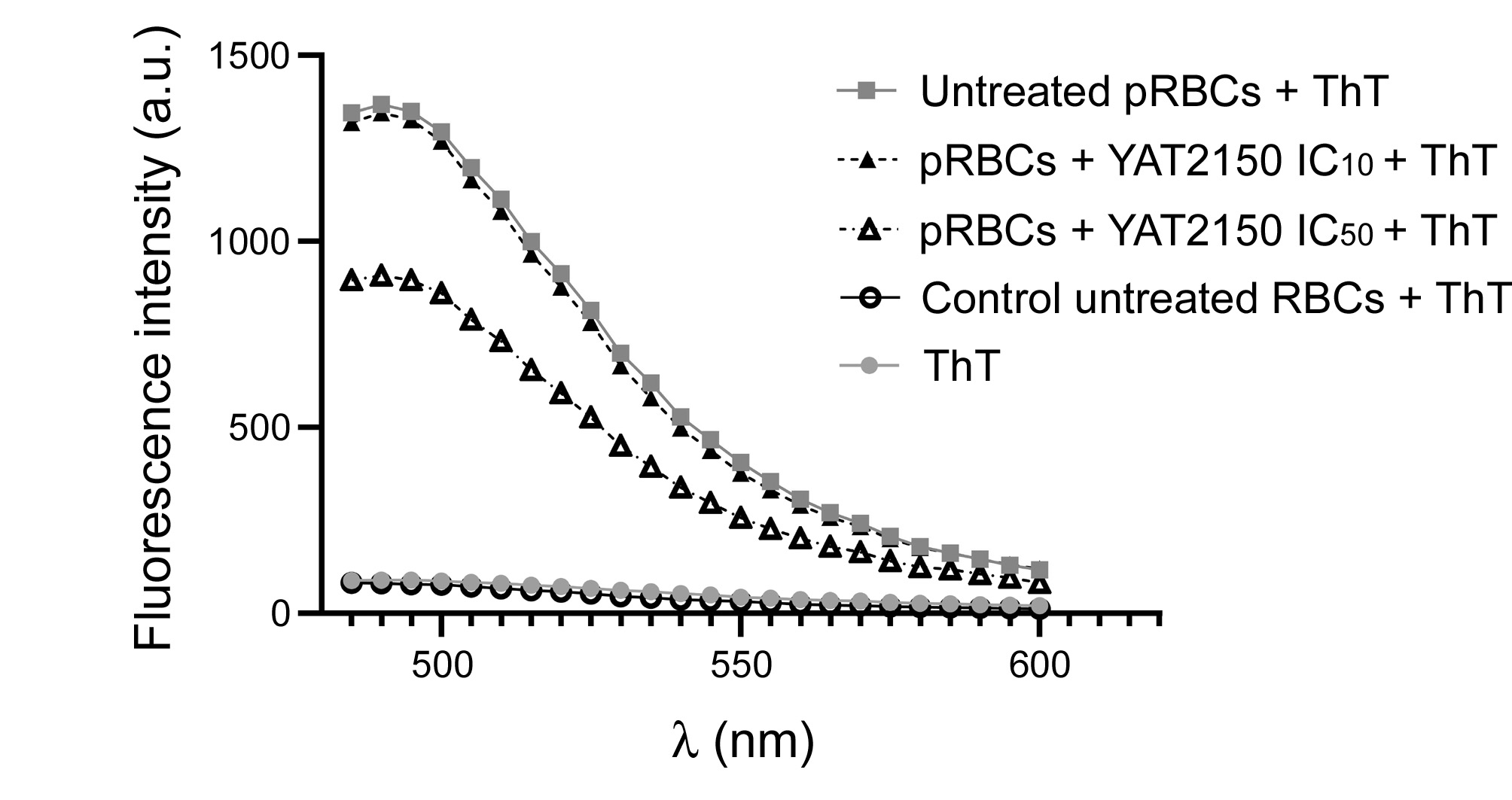


**Figure S11. Determination of protein aggregation in live *P. falciparum* cultures.** ThT fluorescence of *P. falciparum* culture extracts normalized to have equal protein content, either non-treated or treated with YAT2150 at its *in vitro* IC_10_ (27 nM) and IC_50_ (90 nM), for 30 h. A non-parasitized RBC protein extract is shown as reference. The mean fluorescence intensity value of each sample in each wavelength is represented**.**


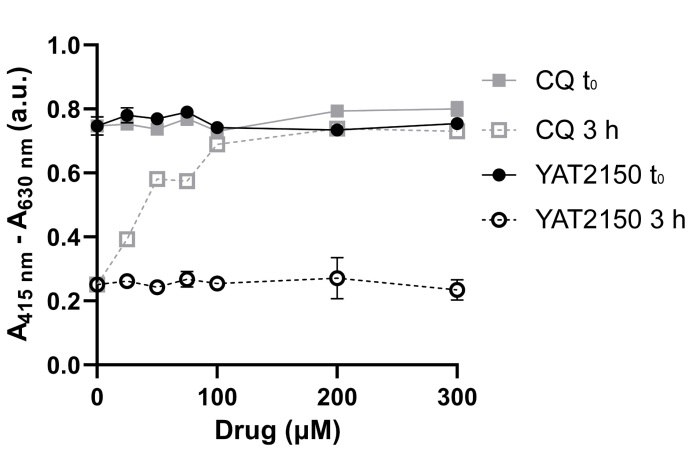


**Figure S12. Hemozoin formation assay.** CQ: chloroquine; a.u.: arbitrary units. Mean ± SD are plotted.

Table S1. Aggregative peptides selected from the pool of 369 proteins resisting dissolution in 0.1% SDS identified in Biosca *et al.*, 2020^1^. In bold are indicated the six peptides selected to be elongated by CPPs.

| Peptide  (aggregation score^2^) | *P. falciparum* proteins containing the peptide (UniProt accession code) | C^3^ |
| --- | --- | --- |
| LQSNIG  (96.3) | Nucleoporin NUP221, putative (Q8I398) | 0.49 |
| NVNIYN  (98.7) | Uncharacterized protein (C0H4L9) | 0.41 |
|  | AP2 domain transcription factor, putative (C0H5G5) | 0.29 |
|  | DNA-binding chaperone, putative (Q8I5N9) | 0.50 |
|  | DNA-(apurinic or apyrimidinic site) lyase (Q8I2Y2) | 0.34 |
|  | Heptatricopeptide repeat-containing protein, putative (Q8IJS9) | N/D^4^ |
| NFNNIYH  (98.3) | AP2 domain transcription factor, putative (C0H5G5) | 0.29 |
| NNFYYNN  (99.0) | AP2 domain transcription factor, putative (C0H5G5) | 0.29 |
| LISFIL  (97.3) | ER membrane protein complex subunit 6, putative (Q8I5R2) | 0.39 |
| LYWIYY  (99.7) | Eukaryotic translation initiation factor 2 subunit A, putative (Q8IJL2) | 0.48 |
| MYVIYV  (99.7) | Eukaryotic translation initiation factor 3 subunit E, putative (Q8I3I5) | 0.55 |
| TIIFIN  (98.3) | Exportin-7, putative (C0H530) | 0.41 |
| GLVFFI  (98.3) | High molecular weight rhoptry protein 2 (C0H571) | 0.70 |
| YLFFIS  (98.7) | AP3 complex subunit delta, putative (C0H4T0) | 0.37 |
|  | Conserved membrane protein, unknown function (Q8I3K4) | 0.43 |
|  | Conserved protein, unknown function (Q8IID6) | 0.29 |

^1^ Biosca A, Bouzón-Arnáiz I, Spanos L, Siden-Kiamos I, Iglesias V, Ventura S, Fernàndez-Busquets X (2020) Detection of protein aggregation in live *Plasmodium* parasites. Antimicrob Agents Chemother 64: e02135-19.

^2^ WALTZ average aggregation score per residue.

^3^ Normalized protein abundance according to PaxDb database.

^4^ No abundance data available.

Table S2. Growth inhibition assay in regular *P. falciparum* cultures of fluorescein-labeled aggregative peptides conjugated to CPPs.

| Peptide sequence | Highest concentration tested (µM) | Parasite growth relative to control (%) ± SEM |
| --- | --- | --- |
| TAT-LQSNIG | 15 | 99.0 ± 11.2 |
| TAT-NVNIYN | 15 | 97.0 ± 5.0 |
| TAT-NFNNIYH | 15 | 93.5 ± 1.0 |
| TAT-NNFYYNN | 7.5 | 100.0 ± 3.3 |
| TAT-LISFIL | 20 | 100.0 ± 4.5 |
| TAT-LYWIYY | 125 | 100.0 ± 1.2 |
| TP2-LQSNIG | 3.5 | 97.5 ± 3.0 |
| TP2-NVNIYN | 3.5 | 100.0 ± 3.8 |
| TP2-NFNNIYH | 50 | 100.0 ± 12.8 |
| TP2-NNFYYNN | 50 | 100.0 ± 4.0 |
| TP2-LISFIL | 20 | 96.4 ± 2.0 |
| TP2-LYWIYY | 125 | 99.1 ± 1.1 |
| LMWP-LQSNIG | 20 | 90.4 ± 6.3 |
| LMWP-NVNIYN | 20 | 89.5 ± 1.8 |
| LMWP-NFNNIYH | 20 | 84.6 ± 4.0 |
| LMWP-NNFYYNN | 200 | 100.0 ± 1.4 |
| LMWP-LISFIL | 20 | 100.0 ± 3.3 |
| LMWP-LYWIYY | 125 | 100.0 ± 6.9 |

Table S3. Growth inhibition assay in ghost RBC-enriched *P. falciparum* cultures treated with 10 µM non-modified aggregative peptides.

| Peptide sequence | Parasite growth relative to control (%) ± SEM |
| --- | --- |
| LQSNIG | 95.3 ± 7.4 |
| NVNIYN | 94.3 ± 24.6 |
| NFNNIYH | 100.0 ± 21.4 |
| NNFYYNN | 89.7 ± 12.7 |
| LISFIL | 100.0 ± 29.4 |
| LYWIYY | 100.0 ± 6.3 |
| MYVIYV | 100.0 ± 31.8 |
| TIIFIN | 100.0 ± 17.5 |
| GLVFFI | 79.4 ± 0.9 |
| YLFFIS | 77.5 ± 12.3 |

**Table S4. Cytotoxicity assay in HUVEC cultures of the aggregative peptides which at 10 µM reduced by > 20% *P. falciparum* growth in ghost pRBC cultures.** The maximum peptide concentration tested was 200 µM.

| Peptide sequence | CC_50_ (µM) ± SEM |
| --- | --- |
| (5/6-FAM)-LMWP-LQSNIG | 77.6 ± 16.0 |
| (5/6-FAM)-LMWP-NFNNIYH | 56.0 ± 1.1 |
| (5/6-FAM)-LMWP-NNFYYNN | 7.9 ± 1.3 |
| (5/6-FAM)-LMWP-LISFIL | 9.0 ± 1.1 |
| GLVFFI | > 200* |
| YLFFIS | > 200** |

* Growth inhibition at 200 µM was 14.9 ± 1.8%.

** Growth inhibition at 200 µM was 22.3 ± 7.9%.

**Table S5. Early stage *P. falciparum* proteins resisting dissolution in 0.1% SDS.** Those found also in late stages are indicated in **bold**.

| UniProtKB code | Protein | Molecular weight (kDa) | Isoelectric point |
| --- | --- | --- | --- |
| A0A5K1K8J3 | Glideome-associated connector | 290.8 | 5.25 |
| A0A5K1K967 | Elongation factor 1-gamma, putative | 47.7 | 7.39 |
| **C0H4K6** | **E3 ubiquitin-protein ligase** | **460.1** | **6.58** |
| C0H4M6 | Regulator of chromosome condensation, putative | 78.8 | 6.89 |
| **C0H4V6** | **14-3-3 protein** | **30.2** | **4.92** |
| C0H577 | Uncharacterized protein | 545.8 | 8.85 |
| **C6KSV0** | **Histone H3** | **15.4** | **11.14** |
| **C6KT18** | **Histone H2A** | **14.1** | **10.29** |
| **O96124** | **Erythrocyte membrane protein 3** | **273.5** | **8.85** |
| **O96258** | **40S ribosomal protein S26** | **12.5** | **10.98** |
| **Q76NM3** | **L-lactate dehydrogenase** | **34.1** | **7.55** |
| **Q7K6A4** | **S-adenylmethionine synthase** | **44.8** | **6.74** |
| **Q8I0P6** | **Elongation factor 1-alpha** | **48.9** | **9.06** |
| Q8I2F2 | PRESAN domain-containing protein | 45.5 | 9.70 |
| Q8I2G1 | Ring-exported protein 1 | 83.0 | 5.59 |
| **Q8I3B0** | **60S ribosomal protein L32** | **15.5** | **11.06** |
| **Q8I492** | **Mature parasite-infected erythrocyte surface antigen** | **168.2** | **4.78** |
| Q8I4R5 | Rhoptry neck protein 3 | 263.0 | 9.20 |
| **Q8I4X0** | **Actin-1** | **41.8** | **5.34** |
| **Q8I5S6** | **Eukaryotic translation initiation factor 3 subunit A** | **166.0** | **6.81** |
| Q8IAK9 | PRESAN domain-containing protein | 147.0 | 6.05 |
| **Q8IAX5** | **40S ribosomal protein S16, putative** | **16.3** | **10.24** |
| **Q8IB24** | **Heat shock protein 70** | **73.9** | **5.67** |
| Q8IDG8 | Membrane associated histidine-rich protein 2 | 15.8 | 7.37 |
| **Q8IE09** | **60S ribosomal protein L23, putative** | **15.0** | **9.86** |
| Q8II97 | Autophagy-related protein 23, putative | 110.0 | 6.60 |
| **Q8IIV1** | **Histone H2B** | **13.1** | **10.26** |
| **Q8IIV2** | **Histone H4** | **11.4** | **11.22** |
| **Q8IJD0** | **Peroxiredoxin** | **43.9** | **9.70** |
| **Q8IKF0** | **Eukaryotic initiation factor 4A** | **45.3** | **5.69** |
| **Q8IKK7** | **Glyceraldehyde-3-phosphate dehydrogenase** | **36.6** | **7.69** |
| **Q8ILV2** | **60S ribosomal protein L10, putative** | **25.2** | **9.95** |
| **Q9TY99** | **Knob-associated histidine-rich protein** | **71.3** | **9.09** |

Table S6. Aggregative peptides identified in *P. falciparum* E3 ubiquitin-protein ligase (C0H4K6).

| Amino acids | Sequence | WALTZ  score | Number of proteins in which the peptide is present |
| --- | --- | --- | --- |
| 3-18 | KYLLFENSQYSYIINS | 95.21 | 1 |
| 116-122 | SFFKIIQ | 94.98 | 1 |
| 274-281 | TEIIINSE | 97.99 | 1 |
| 314-321 | KFFLSNIN | 96.57 | 1 |
| 323-346 | VDVKYITIIYTATCCIYTILDIYP | 95.40 | 1 |
| 357-363 | EAVYILN | 97.99 | 1 |
| 377-382 | VILKIF | 94.31 | 1 |
| 409-414 | YNVNIQ | 96.98 | 2 |
| 417-429 | IFFCIIQMINNIT | 95.86 | 1 |
| 441-452 | YCNIFINFFHYH | 97.07 | 1 |
| 455-461 | HILNIIC | 96.46 | 1 |
| 678-687 | IESIYNINIR | 97.99 | 1 |
| 702-721 | NLYAFMETFYIISILVHYSN | 97.16 | 1 |
| 748-763 | NNFLIIYILFTLYSFL | 96.20 | 1 |
| 787-796 | FYENIGEFIN | 92.64 | 1 |
| 901-907 | NIYYIYE | 97.52 | 1 |
| 917-936 | LCILICLSVFISLYKISLTC | 94.70 | 1 |
| 948-953 | YIFSYN | 98.33 | 1 |
| 986-992 | FTLLILK | 93.98 | 1 |
| 1023-1031 | GGLLFNITH | 97.73 | 1 |
| 1043-1048 | VLLHIV | 94.31 | 1 |
| 1076-1090 | KIYNIIFMYFYDIFN | 93.87 | 1 |
| 1108-1112 | KDLLF | 96.32 | 10 (4 putative) |
| 1122-1132 | VFLEYSELFFN | 97.08 | 1 |
| 1145-1151 | NICSYFK | 93.31 | 1 |
| 1182-1194 | ILCYYYLIYLFSN | 98.12 | 1 |
| 1202-1208 | SCIYFYQ | 95.13 | 1 |
| 1216-1226 | YFFLYQSLLYG | 99.33 | 1 |
| 1266-1277 | RLYIFLYALLFV | 97.94 | 1 |
| 1325-1345 | LVVKIFDSYHYYLIINNLSFK | 97.60 | 1 |
| 1387-1392 | YYDYIN | 92.98 | 2 |
| 1462-1469 | QDFSYNVY | 96.99 | 1 |
| 1647-1651 | KVVNI | 96.99 | 9 (2 putative) |
| 1730-1747 | EYNLYNYFNNNRYINYIP | 96.77 | 1 |
| 1752-1767 | YENLFNESINNNLTID | 96.99 | 1 |
| 1775-1780 | MYNNYN | 98.33 | 3 |
| 1796-1801 | NVSIFG | 93.98 | 2 |
| 1837-1845 | KQNNFNDYF | 97.66 | 1 |
| 1935-1944 | GSFNIFETFN | 96.49 | 1 |
| 2017-2028 | IFCNIENFYIYN | 97.80 | 1 |
| 2049-2059 | WNNNYNINNNN | 96.32 | 1 |
| 2118-2125 | NNNIYSLN | 96.32 | 3 |
| 2127-2134 | NNNIYSLN | 96.32 | 3 |
| 2138-2143 | NSVNYK | 92.64 | 2 |
| 2152-2157 | NNSLFM | 92.64 | 1 |
| 2198-2209 | SVSNYVDWVTYK | 96.66 | 1 |

Table S7. *P. falciparum* proteins in which the peptides KDLLF and KVVNI are present.

| Peptide | Proteins (UniProt accession code) | C^1^ |
| --- | --- | --- |
| KDLLF | E3 ubiquitin-protein ligase (C0H4K6) | 0.49 |
|  | conserved protein, unknown function (Q8IJE8) | N/D^2^ |
|  | conserved membrane protein, unknown function (A0A143ZZV5) | N/D^2^ |
|  | DNA polymerase epsilon catalytic subunit A, putative (C6KTD8) | 0.35 |
|  | conserved protein, unknown function (Q8IKF7) | N/D^2^ |
|  | ATP-dependent RNA helicase DHR1, putative (Q8IET8) | 0.28 |
|  | Cg1 protein (Q8IBZ8) | 0.39 |
|  | tetratricopeptide repeat protein, putative (Q8IM60) | 0.41 |
|  | NADPH-cytochrome P450 reductase, putative (Q8IKX3) | 0.58 |
|  | palmitoyltransferase DHHC12, putative (A0A146M427) | N/D^2^ |
| KVVNI | E3 ubiquitin-protein ligase (C0H4K6) | 0.49 |
|  | conserved protein, unknown function (Q8ILS9) | 0.29 |
|  | conserved protein, unknown function (Q8IIT8) | 0.31 |
|  | sodium/hydrogen exchanger (Q8IET0) | 0.37 |
|  | *S*-adenosylmethionine decarboxylase/ornithine decarboxylase (Q8IJ77) | 0.42 |
|  | kinesin-like protein, putative (C0H4S3) | 0.20 |
|  | conserved protein, unknown function (Q8IJF2) | N/D^2^ |
|  | splicing factor 1 (Q8IE99) | 0.47 |
|  | glutamate-tRNA ligase, putative (Q8IDK7) | 0.70 |

^1^ Normalized protein abundance according to PaxDb database.

^2^ No abundance data available.

Table S8. Growth inhibition assay in ghost RBC-enriched *P. falciparum* cultures treated with 10 µM KDLLF and KVVNI peptides.

| Peptide | Parasite growth relative to control (%) ± SEM |
| --- | --- |
| KDLLF | 100.0 ± 1.7 |
| FAM-KDLLF | 100.0 ± 14.1 |
| KVVNI | 88.2 ± 14.2 |
| FAM-KVVNI | 94.0 ± 9.6 |

**Table S9. *In vitro* toxicity in HUVEC cultures of amyloid pan-inhibitors.**

| Compound | CC_50_ (µM) ± SEM |
| --- | --- |
| HUP5ANTRA | 76.2 ± 21.5 |
| HUP7ANTRA | 29.0 ±1.5 |
| HUP10ANTRA | 84.7 ± 13.3 |
| HUP7TH | 7.8 ± 3.4 |
| HUP8TH | 4.9 ± 1.3 |
| HUP9TH | 4.9 ± 1.0 |
| HUPH10TH | 3.4 ± 0.1 |
| HUPNTH | 3.4 ± 1.0 |
| HUPNTCl | 6.3 ± 0.5 |
| DP128 | 49.0 ± 0.1 |
| DONE3TCl | 12.6 ± 2.2 |
| YAT2150 | 3.4 ± 0.5 |
